# Lipidomic Signature of Patients with Familial Hypercholesterolemia Carrying Pathogenic Variants Unveils a Cue of Increased Cardiovascular Risk

**DOI:** 10.3390/ijms262110688

**Published:** 2025-11-03

**Authors:** Giulia De Simone, Maria Donata Di Taranto, Debora Paris, Martina Ferrandino, Marco Andolfi, Annalaura Iodice, Giovanna Cardiero, Carmine De Luca, Luigi Junior Valletta, Ilenia Lorenza Calcaterra, Gabriella Iannuzzo, Matteo Nicola Dario Di Minno, Giuliana Fortunato, Adele Cutignano

**Affiliations:** 1 Consiglio Nazionale delle Ricerche (CNR), Istituto di Chimica Biomolecolare (ICB), Via Campi Flegrei 34, 80078 Pozzuoli, Italy; giuliadesimone@cnr.it (G.D.S.); debora.paris@cnr.it (D.P.); annalauraiodice@cnr.it (A.I.); 2 Dipartimento di Medicina Molecolare e Biotecnologie Mediche, Università degli Studi di Napoli Federico II, Via Pansini 5, 80131 Napoli, Italy; mariadonata.ditaranto@unina.it (M.D.D.T.); martina.ferrandino@unina.it (M.F.); giovanna.cardiero@unina.it (G.C.); fortunat@unina.it (G.F.); 3 CEINGE Biotecnologie Avanzate Franco Salvatore, Via Gaetano Salvatore 486, 80145 Napoli, Italy; 4 Dipartimento di Medicina Clinica e Chirurgia, Università degli Studi di Napoli Federico II, Via Pansini 5, 80131 Napoli, Italy; deluca.carmine.medicina@gmail.com (C.D.L.); vallettaluigijunior@gmail.com (L.J.V.); ilenialorenza.calcaterra@unina.it (I.L.C.); gabriella.iannuzzo@unina.it (G.I.); matteo.diminno@unina.it (M.N.D.D.M.); 5 Centro di Riferimento Regionale di Diagnosi e Terapia Delle Dislipidemie Nell’adulto, Azienda Ospedaliera Universitaria Federico II, Via Pansini 5, 80131 Napoli, Italy

**Keywords:** dyslipidemia, lipidomics, liquid chromatography-mass spectrometry, nuclear magnetic resonance, sphingomyelins

## Abstract

Familial Hypercholesterolemia (FH) is a common genetic disorder characterized by elevated LDL-cholesterol levels and an increased risk of premature cardiovascular disease. While pathogenic variants in *LDLR*, *APOB*, and *PCSK9* are well-established causes, a substantial proportion of clinically suspected FH cases do not carry either pathogenic variants or rare variants of uncertain significance in these genes (FH/V−/USV−). This study aimed to characterize the metabolome/lipidome of genetically confirmed heterozygous FH (HeFH) patients compared to FH/V−/USV−, seeking to identify specific alterations associated with genetic status and phenotypic variability. Untargeted high-resolution mass spectrometry (UHPLC-Q-Exactive-MS)-based lipidomics and nuclear magnetic resonance-based metabolomics were performed on plasma samples of FH patients (n = 20 HeFH and n = 19 FH/V−/USV−) towards healthy controls (n = 22). PLS-DA analysis revealed group-level separation, suggesting differences in the circulating metabolome/lipidome. As expected, most of identified lipid classes were higher in both FH groups compared to normolipidemic controls. Notably, significant lipids (VIP > 1, *p* < 0.05) showed potential in distinguishing HeFH and FH/V−/USV− patients, particularly sphingomyelins. These data were confirmed by multivariable regression analysis controlling for age, sex, and lipid-lowering therapy as well as by ROC analysis. The evidence of a distinct lipidome signature in the HeFH subgroup may relate to the increased cardiovascular risk of HeFH patients compared to patients without pathogenic variants.

## 1. Introduction

Familial Hypercholesterolemia (FH) is an autosomal dominant disorder of lipoprotein metabolism that is associated with increased low-density lipoprotein cholesterol (LDL-C) levels from birth onward, leading to premature atherosclerosis and coronary artery disease (CAD) [1,2,3]. The disease is caused by different pathogenic variants in the genes coding for LDL receptor (*LDLR*), apolipoprotein B (*APOB*), and proprotein convertase subtilisin/kexin type 9 (*PCSK9*) [4,5,6].

There are two forms of the disease: the most frequent is heterozygous FH (HeFH), with a prevalence of ~1:200 [7], while homozygous FH (HoFH) has a lower prevalence (~1:300,000) [8,9,10]. However, a conspicuous percentage of patients (20–30%) with a clinical suspicion of FH do not carry any pathogenic variants (FH/V−) [2]. In recent years, the growing number of identified FH-associated variants has complicated genetic diagnosis, with a significant proportion of these variants being classified as of ‘uncertain significance’ (USV) or having conflicting evidence, a factor that often hampers a clear interpretation of genetic results [11].

Recently, the different phenotypes associated with the presence of a genetic variant causative of FH have aroused great interest, as the detection of a pathogenic variant in one of the candidate genes is a cardiovascular risk factor independently of the LDL-C levels [12,13,14]. However, the metabolomic profile associated with the presence of a pathogenic variant compared to its absence remains unclear and not fully investigated. In this study, we aimed to investigate the differences in the lipidome and metabolome profiles of patients with genetically confirmed HeFH compared with those without genetic variants, i.e., neither pathogenic nor USV (FH/V−/USV−). In particular, we evaluated specific lipid and metabolite alterations that could contribute to the phenotypic variability observed in FH and its broader metabolic implications. These findings may have important clinical implications, as a deeper understanding of lipid dysregulation in FH could support an early diagnosis, as well as in the development of more personalized therapeutic strategies around optimizing lipid-lowering interventions based on the patient’s specific metabolic profile.

## 2. Results

### 2.1. Characteristics of the Study Population

Sixty-one individuals were included in the study: 39 patients with clinical FH, comprising 20 HeFH patients, and 19 without pathogenic or uncertain significance variants (FH/V−/USV−), along with 22 healthy individuals used as controls. The demographic and clinical characteristics of the study population are summarized in Table 1.

There were no significant differences in sex distribution among the three groups (*p* = 0.7828). However, FH/V−/USV− patients were significantly older than both healthy controls and HeFH patients (58.5 ± 13.1, 46.6 ± 12.0, and 46.1 ± 14.2 years, respectively; *p* = 0.0047).

As expected, compared to normolipidemic controls, both FH groups showed elevated lipid levels. Total cholesterol was significantly higher in HeFH patients than in controls (*p* = 0.0242), as were non-HDL cholesterol (non-HDL-C) (*p* = 0.0026) and triglycerides (*p* = 0.0071). LDL-C levels tended to be higher in the HeFH group compared to healthy controls and FH/V−/USV− patients (131.2 ± 52.4, 94.5 ± 14.3 and 104.8 ± 61.3 mg/dL, respectively, *p* = 0.0467).

No significant differences between groups were observed in HDL-C levels (*p* = 0.1349) or in lipid-lowering therapy use between HeFH and FH/V−/USV− patients (*p* = 0.1005), although high-intensity treatments were more frequent in the HeFH group.

These data reflect the expected lipid alterations in hypercholesterolemic patients compared to healthy controls and highlight that FH/V−/USV− and HeFH showed an overlapping profile of lipid biomarkers used as reference in routine biochemical analyses.

### 2.2. Distinct Lipidomic Signatures in FH Groups and Healthy Controls

An ultrahigh-performance liquid chromatography coupled with high resolution mass spectrometry lipidomic analysis was performed to seek differences in the plasma lipidome between patients with HeFH, FH/V−/USV− patients, and controls.

**Figure 1:**
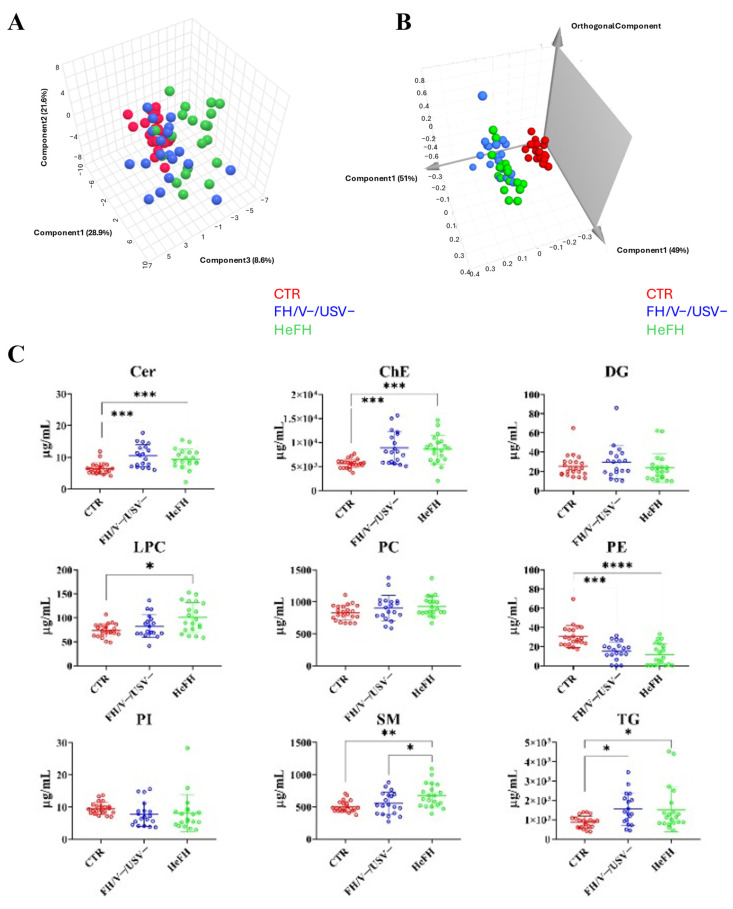
(**A**) PLS-DA 3D score plot performed on the total number of lipids identified; (**B**) OPLS-DA 3D score plot obtained from NMR metabolic profiles of plasma samples; (**C**) scatter plot representing the concentration (µg/mL plasma) of the detected lipid classes in hyperlipidaemic patients and healthy controls (CTR) (Kruskal–Wallis test, * *p* < 0.05, ** *p* < 0.01, *** *p* < 0.001, **** *p* < 0.0001).

The results from the PLS-DA analysis (Figure 1A) revealed a clear separation between the groups based on their lipid levels, highlighting specific lipid species that distinguish patients and healthy individuals. In particular, the first and second principal components (PC1 and PC2) effectively distinguished healthy controls from both patient groups (HeFH and FH/V−/USV−). Notably, the third principal component (PC3) contributed to the separation between the HeFH and FH/V−/USV− groups, suggesting distinct lipidomic signatures associated with the presence of a pathogenic variant.

When the data were aggregated by lipid class, significant alterations were revealed in the patient groups compared to healthy controls (Figure 1C; Supplementary Material Table S2; Kruskal–Wallis test, *p* < 0.05). Across all samples, the most abundant lipid classes were cholesteryl esters (ChE, ranging between 4.98 and 11.82 mg/mL), triglycerides (TG, ranging between 0.62 and 2.16 mg/mL) and phosphatidylcholines (PC, ranging between 0.74 and 1.02 mg/mL), which was as expected. The least abundant classes were phosphatidylinositol (PI, ranging between 4.49 and 10.43 µg/mL) and ceramides (Cer, ranging between 5.33 and 17.69 µg/mL).

Stratifying by genetic status, both HeFH and FH/V−/USV− patients showed median ChE (8.70 and 8.08 mg/mL, respectively) and TG (1.13 and 1.36 mg/mL, respectively) higher than healthy controls (5.68 mg/mL and 0.90 mg/mL, respectively; Kruskal–Wallis test, *p* < 0.05). Moreover, lysophosphatidylcholines (LPC) and sphingomyelins (SM) were all significantly elevated only in the HeFH (93.33 µg/mL and 648.50 µg/mL, respectively) group compared to the control group (71.98 µg/mL and 480.83 µg/mL, respectively; Kruskal–Wallis test, *p* < 0.05). A direct comparison between the two groups of patients showed that SM levels were significantly higher in the HeFH group compared to the FH/V−/USV− group (Kruskal–Wallis test, *p* < 0.05). No differences were observed in diacylglycerols (DG), phosphatidylcholines (PC), or phosphatidylinositol (PI) between all analyzed groups.

**Figure 2:**
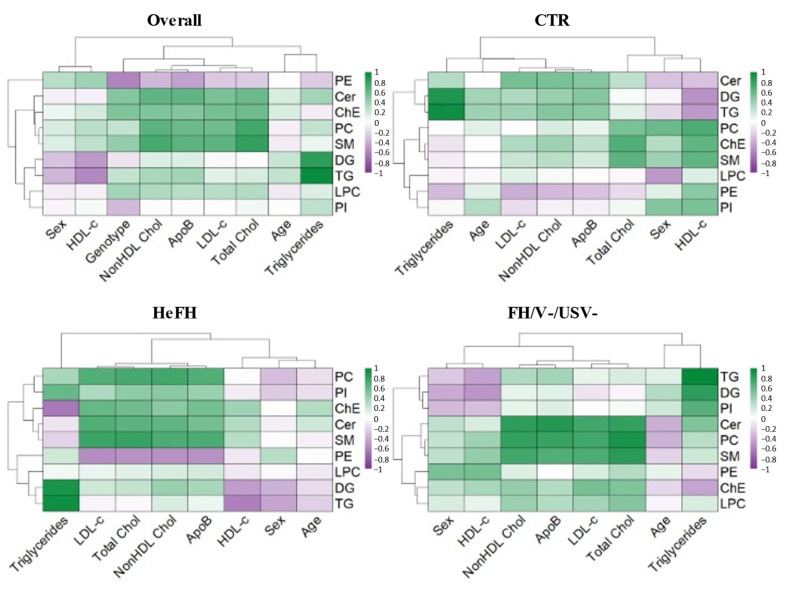
Faceted heatmap displaying Spearman correlations between lipid classes and clinical data in the overall cohort, healthy individuals (CTR), HeFH, and FH/V−/USV−. Color saturation corresponds to the value of Spearman’s correlation coefficient.

Spearman correlation analysis was conducted between clinical variables (sex, age, genotype, and standard lipid profile) and the concentration of lipid classes, measured by LC-MS in the entire study population (n = 61) and stratified by group (healthy controls, HeFH, and FH/V−/USV−) (Figure 2; Supplementary Material Table S3).

Significant correlations were observed between several lipid classes and genotype, coded as follow: healthy controls as 0, FH/V−/USV− as 1, and HeFH as 2. In particular, Cer (r = 0.48, *p* < 0.001), ChE (r = 0.51, *p* < 0.0001), LPC (r = 0.36, *p* = 0.0044), and SM (r = 0.42, *p* = 0.001) all showed positive associations with the presence of pathogenic variants. Conversely, PE showed a strong inverse correlation (r = −0.59, *p* < 0.0001). In addition, LDL-C and ApoB levels were positively correlated with each other (r = 0.85, *p* < 0.0001) as well as with various lipid classes, including PC (r = 0.56, r = 0.58, *p* < 0.0001, respectively), SM (r = 0.65, r = 0.65, *p* < 0.0001, respectively), and Cer (r = 0.53, r = 0.63, *p* < 0.0001, respectively).

Stratified analyses revealed stronger associations in the HeFH and FH/V−/USV− groups compared to controls, in which correlations were generally weak and dispersed. In HeFH patients, LDL-C, ApoB, and total cholesterol levels were significantly correlated with SM (r = 0.78, 0.71, and 0.78, *p* < 0.001, respectively), PC (r = 0.69, 0.68, and 0.73, *p* < 0.001, respectively), and Cer (r = 0.68, 0.74, and 0.65, *p* < 0.001, respectively). Similar correlations were observed in patients without pathogenic variants (FH/V−/USV−): LDL-C, ApoB, and total cholesterol were correlated with Cer (r = 0.75, 0.86, and 0.80, *p* < 0.001, respectively), SM (r = 0.71, 0.69, and 0.84, *p* < 0.001, respectively), and PC (r = 0.69, 0.78, and 0.91, *p* < 0.001, respectively). These findings suggest a lipidomic signature that is more tightly associated with lipid levels in genetically and clinically suspected FH patients than in healthy controls.

### 2.3. Distinct Metabolic NMR-Based Profiles in FH Patients and Healthy Individuals

The Nuclear Magnetic Resonance (NMR)-based metabolic profiles of plasma samples from HeFH patients, FH/V−/USV− patients, and healthy controls were analyzed, searching for specific molecular differences using OPLS-DA regression. The model score plot (Figure 1B) demonstrated a clear separation between the control group and the patient groups (HeFH and FH/V−/USV− patients) along with the first principal component (PC1). Furthermore, a trend for separation between the two FH groups was observed along with the second principal component (PC2). Although there was some overlap between the two patient groups, the HeFH one tended to be slightly detached at positive values for the second component, while the centroid of the FH/V−/USV− group appeared more detached at negative coordinates (Supplementary Material Figure S2A). Inspection of the corresponding loadings plot (Supplementary Material Figure S2B) identified the NMR signals, and subsequently the metabolites responsible for this discrimination. The analysis revealed that several amino acids, glucose, and organic acids were present at statistically higher levels in both FH groups compared to the healthy controls. Specifically, glutamate, lysine, threonine, proline, and isoleucine concentrations were elevated in both HeFH and FH/V−/USV− groups versus the healthy controls, whereas no differences were observed between the two FH groups. Regarding organic acids, lactate, 2-hydroxybutyrate, and 3-hydroxybutyrate showed higher concentrations in both FH groups compared to healthy controls. Conversely, glutamine, tyrosine, and acetate levels, together with glucose, were found to be lower in both patient groups compared to the healthy controls. All reported metabolite variations were statistically significant when comparing the patient groups with the healthy controls (Table 2). Although not statistically significant, higher levels of leucine, isoleucine, glucose, and lactate were observed in the FH/V−/USV− group compared to the HeFH one.

### 2.4. Lipidomic Signatures of FH Groups

Elucidating the specific lipidomic signature of the HeFH and FH/V−/USV− groups aimed to identify potential biomarkers of the increased CVD risk observed in carriers of a FH pathogenic variant (Table 3).

We observed that SM, in particular SM (d38:4), SM (d43:2), SM (d44:4), and SM (d32:0), were significantly more abundant in HeFH patients compared to the other FH group (*p*-values ranging from 0.0007 to 0.0128). SM (d32:0) and SM (d38:4) were the less abundant among the statistically significant SM (0.31–0.78 and 0.44–0.93 µg/mL, respectively). In addition to sphingomyelins, PC (18:0/20:4) and LPC (20:4) were also significantly higher in the HeFH group compared to the FH/V−/USV− one (102.60 µg/mL vs. 80.53 µg/mL; *p*-value = 0.0014, FC = 1.37 and 5.51 µg/mL vs. 4.23 µg/mL; *p*-value = 0.0151, FC = 1.30, respectively).

Conversely, PE (18:0p/18:2) and Cer (d18:1/23:0) were higher in the FH/V−/USV− group (1.17 µg/mL and 1.02 µg/mL, respectively) compared to HeFH patients (0.38 µg/mL and 0.73 µg/mL, respectively) (*p*-value = 0.0275, FC = −3.09; *p*-value = 0.0283, FC = −1.41, respectively).

Multivariable linear regression analysis was performed to assess the independent associations between lipid levels and genotype, lipid lowering therapy, sex, and age. To this end, two patients were excluded due to PCSK9 inhibitors (PCSK9i) administration, since previous results from some of the authors of this study [15] showed a change in specific lipid species following therapy with PCSK9i; one additional patient was excluded because of lactation status, which led to the temporary discontinuation of her therapy, in order to exclude the possibility of other confounders. The results showed that different lipid species were influenced by distinct factors, with genotype and therapy emerging as the most relevant predictors for certain metabolites, while sex was a significant factor for others (Table 4). In particular, the presence of a pathogenic variant was significantly associated with five lipids belonging to SM or PC classes. Patients carrying a pathogenic variant had higher levels of SM (d38:4) (β = 0.22, *p* = 0.0059), SM (d43:2) (β = 2.99, *p* = 0.0003), SM (d44:4) (β = 2.25, *p* = 0.0118), SM (d32:0) (β = 0.22, *p* = 0.0084), and PC (18:0/20:4) (β = 21.51, *p* = 0.0193) than FH/V−/USV− ones. This association was also confirmed considering the whole lipid class, where total SM concentrations were significantly higher in HeFH patients compared to FH/V−/USV− ones (β = 190.13; *p* = 0.0256) independently of lipid-lowering therapy, sex, or age.

Lipid-lowering therapy was significantly associated with two lipids: LPC(20:4) (β = 1.45, *p* = 0.0134) and PC (18:0/20:4) (β = 20.18 *p* = 0.0180). This suggests that the effect of the therapy may be particularly relevant for these lipid species.

Sex was significantly associated with PE (18:0p/18:2) (β = 0.60, *p* = 0.0277), with higher levels observed in males. No significant associations were found between age and lipid levels (all *p* > 0.05).

Although some lipids showed significant differences between groups in unadjusted comparisons (Kruskal–Wallis test), their associations lost significance in the multivariable regression model. This suggests that their initial differences may have been influenced by other covariates, such as therapy or sex, rather than by genotype alone.

### 2.5. Correlation of Lipidomic Profile with Clinical and Biochemical Data in FH Groups

**Figure 3:**
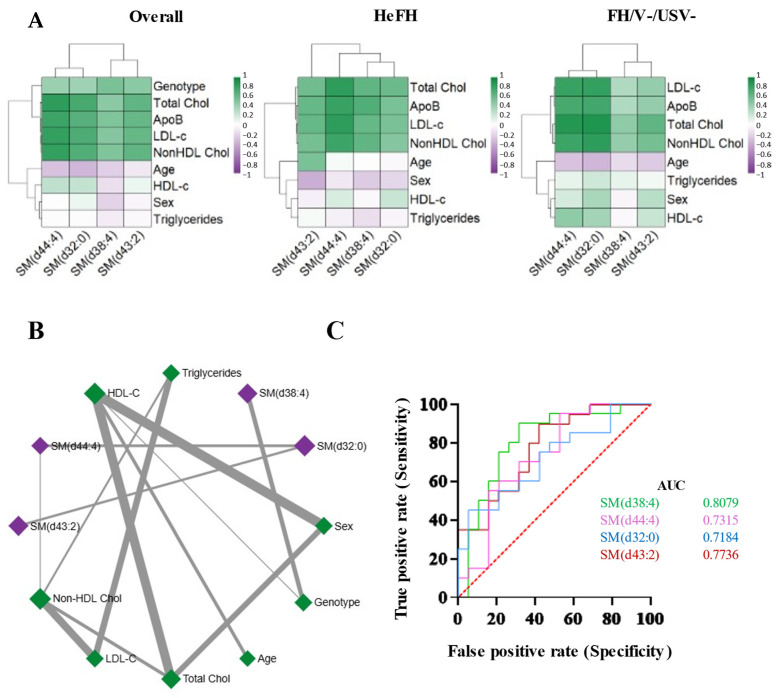
(**A**) Faceted heatmap displaying Spearman correlations between significantly altered SM and clinical data in the patient cohorts, HeFH, and FH/V−/USV−. Color saturation corresponds to the value of Spearman’s correlation coefficient. (**B**) The Debiased Sparse Partial Correlation (DSPC) network used to select the densest lipid–lipid, lipid–biochemical, and biochemical–biochemical data connection subnetworks. (**C**) Receiver Operating Characteristic (ROC) curves illustrating the discriminatory power of the four sphingomyelins in distinguishing between HeFH and FH/V−/USV− patients. Each Area Under the Curve (AUC) provides a measure of discrimination ability.

Among all lipids significantly associated with the genotype in the adjusted model, SM demonstrated the strongest discriminatory power. Moreover, among the SM species significantly altered between HeFH and FH/V−/USV− patients, several showed consistent correlations with biochemical markers across both groups (Figure 3A). Interestingly, no relevant correlations were observed between biochemical data and SM in healthy controls (Supplementary Material, Figure S3). SM (d38:4) was the only species correlated with the genotype when considering the overall groups (r = 0.53, *p* < 0.001). Instead, SM (d44:4) and SM (d32:0) were strongly and positively correlated with LDL-C (r = 0.79 and 0.81, respectively; both *p* < 0.001) and total cholesterol (r = 0.88 and 0.88; *p* < 0.001) in FH/V−/USV− patients, and similarly in HeFH (r = 0.84 and 0.52 with LDL-C; r = 0.82 and 0.59 with total cholesterol). These SM species were also positively associated with non-HDL-C in both groups (SM (d44:4): r = 0.77, *p* < 0.001 in HeFH, r = 0.77, *p* < 0.001 in FH/V−/USV−).

Conversely, other SM species revealed divergent associations. SM (d38:4) was significantly correlated with LDL-C (r = 0.60, *p* = 0.005), total cholesterol (r = 0.62, *p* = 0.004), and non-HDL-C (r = 0.63, *p* = 0.003) in HeFH, but showed only modest or nonsignificant correlations in FH/V−/USV− patients (r = 0.33–0.45, *p* > 0.05). Similarly, SM (d43:2) was correlated with total cholesterol and non-HDL-C only in HeFH (r = 0.57 and 0.55, respectively), with no comparable associations in FH/V−/USV− patients.

Moreover, a correlation network was built using the Debiased Sparse Partial Correlation (DSPC) network function within MetaboAnalyst 6.0 software. In particular, the identified dense subnetwork showed 12 nodes and 13 edges (Figure 3B). The nodes are input parameters (lipids and demographic data) and the edges represent measures of their association, with thicker edges characterized by lower p-values associated within these connections. The analysis confirmed SM (d38:4) as the only lipid species correlated with the presence of a pathogenic variant.

The ROC curve analysis (Figure 3C; Supplementary Material, Table S4) demonstrated that all four sphingomyelin species had AUC values greater than 0.7, confirming their specific association with the genetic status of patients. The combined analysis of sphingomyelins also yielded an AUC > 0.6, reinforcing the robustness of sphingolipid metabolism as a distinguishing feature of HeFH.

## 3. Discussion

FH is a genetic disorder characterized by elevated LDL-C levels, primarily due to the presence of pathogenic variants in genes related with the LDL receptor function and cholesterol clearance. In this study, we used both untargeted mass spectrometry-based lipidomics and NMR-based metabolomics approaches to assess the plasma profiles in patients with HeFH characterized by pathogenic variants in the *LDLR* gene that are known to impair LDL clearance and result in elevated cholesterol levels, in parallel with FH/V−/USV− patients for whom the molecular bases of FH are not related to a specific pathogenic variant and/or remain unclear. Hence, the annotation FH/V−/USV− in this study identifies a hypercholesterolemic population without genetic confirmation of FH and even without potential pathogenic variants still classified as USV. The study was designed to provide a comprehensive comparison of the lipidome and metabolome in these two distinct patient cohorts alongside a control group, thereby elucidating potential differences in key lipid metabolism processes and clarifying the effect of genetic background in hypercholesterolemia.

Although not significant, a trend of higher levels of LDL-C was observed in patients with HeFH compared to the FH/V−/USV− group (*p* = 0.08) and healthy controls (*p* < 0.05). We observed a similar read-out about conventional lipids measurement in blood samples (i.e., total cholesterol, triglycerides, HDL-C, LDL-C, non-HDL-C) in hypercholesterolemia with known genetic alteration and without a specific pathogenic variant, which is consistent with previous findings [16] (Table 1). As expected, both the HeFH and FH/V−/USV− groups exhibited significantly elevated total cholesterol levels compared to the healthy controls (*p* < 0.05), while triglyceride levels were significantly increased only in the FH/V−/USV− group (*p* < 0.01) (Table 1).

These data were confirmed by untargeted MS lipidomic analysis of the three groups that highlighted the alteration of Cer, ChE, LPC, SM, and TG in HeFH and/or FH/V−/USV− patients compared to the healthy controls (*p* < 0.05; FC > 1.30) (Figure 1). Complementing MS analysis, an NMR-based approach highlighted specific alterations in the plasma metabolite profiles of patients compared to the healthy controls. Both FH groups showed elevated levels of several amino acids (glutamate, lysine, threonine, proline, and isoleucine), glucose, and organic acids (lactate, 2-hydroxybutyrate, and 3-hydroxybutyrate) when compared to healthy controls (Table 2). In contrast, glutamine, tyrosine, and acetate levels were consistently lower in both patient groups compared to healthy individuals. These findings are in line with previous reports highlighting that hypercholesterolemia influences systemic metabolism beyond lipid pathways [17,18,19].

**Figure 4:**
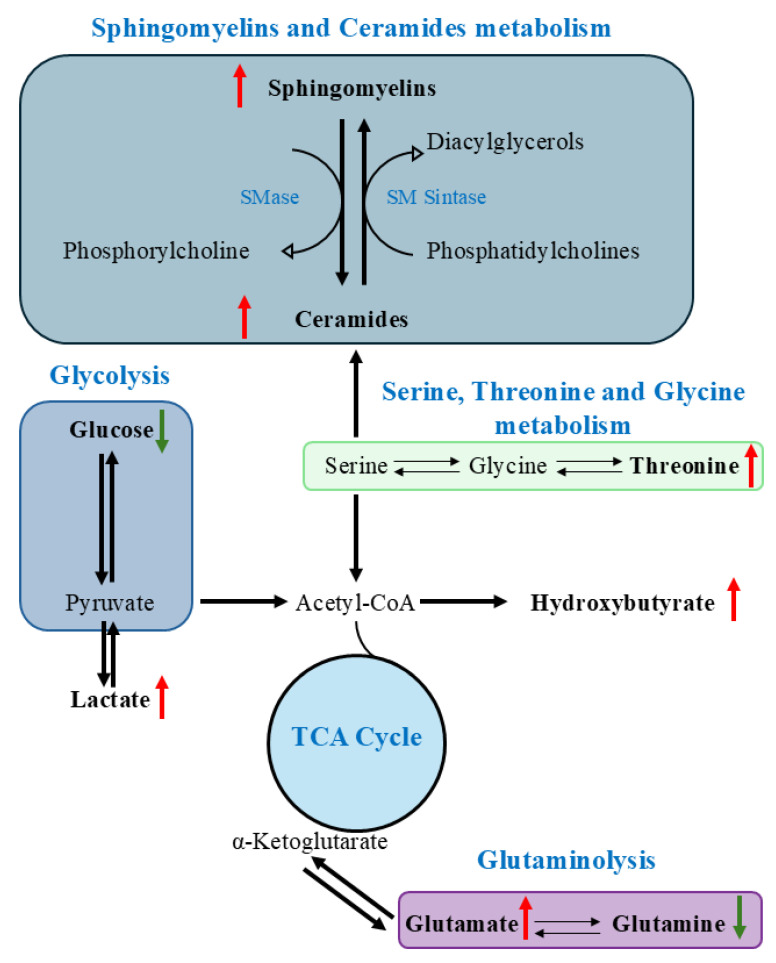
Overview of altered metabolic pathways observed in FH patients, highlighting key lipid and energy metabolism interconnections (red arrow: metabolite/lipid levels higher in FH groups compared to CTR; green arrow: metabolite/lipid levels lower in FH groups compared to CTR).

Overall, the alterations in polar metabolites (amino acids, organic acids and glucose) and central carbon metabolism can be linked to the observed accumulation of SM and Cer from MS lipidomic data, as depicted in Figure 4. In detail, the significant alterations in amino acid metabolism, characterized by increased glutamate and threonine alongside decreased glutamine, reflect imbalances in cellular energy and redox states. Of particular interest, glutamine—a key amino acid for cardiovascular function—was consistently reduced in both FH groups, while glutamate was increased. This glutamine/glutamate imbalance has been associated with increased cardiovascular risk and poor endothelial function [20,21,22,23,24,25]. Moreover, elevated isoleucine and other branched-chain amino acids (BCAAs) in FH patients support previous evidence linking BCAAs to cardiometabolic risk and lipid dysregulation [26,27]. The elevated levels of 3-hydroxybutyrate and 2-hydroxybutyrate in FH patients accompanied by reduced glucose suggest an increased reliance on alternative energy substrates, indicating an altered balance in glucose utilization or impaired oxidative phosphorylation. The co-occurrence of elevated lactate also points towards increased glycolytic flux or a shift towards anaerobic metabolism, possibly due to mitochondrial dysfunction. In fact, cholesterol accumulation has been shown to impair mitochondrial respiration and increase oxidative stress, forcing cells to rely on glycolysis for energy, a process previously associated with atherosclerotic plaque development and cardiovascular risk [28,29].

Although NMR metabolomics highlighted consistent metabolic changes in all FH patients compared to healthy controls, it did not allow us to identify a distinctive signature of HeFH and FH/V−/USV− groups. Nevertheless, while not statistically relevant, altered levels of a few metabolites, as mentioned above, were observed in the FH/V−/USV− group compared to the HeFH one, delineating a clear trend. These potential associations with the genetic status of FH could benefit from a metabolomics investigation in a larger population.

Other studies in FH were carried out in clinically diagnosed FH patients by performing an NMR-metabolomic evaluation of plasma metabolites, focusing the lipid analysis on triglycerides and LDL-C via the Nightingale platform [30,31]. In particular, a data-driven analysis was conducted to clusterize hypercholesterolemic patients with the aim of finding metabolic features associated with the presence of pathogenic variants [30]. The study did not identify metabolic clusters univocally associated with/without genetically confirmed hypercholesterolemia, underlining the complexity of the pathology and the putative occurrence of minor, combined, or unknown gene variants. 

In our study, we adopted an untargeted lipidomic approach to perform a comprehensive analysis, evaluating the levels of lipids in terms of both lipid classes and individual species, beyond routinely monitored lipids that included LDL-C, HDL-C, non-HDL-C and triglycerides. While the majority of lipids showed significant difference between either HeFH or FH/V−/USV− groups versus healthy controls but not between the two groups of patients, we observed significantly higher levels of total SM in patients with HeFH compared to FH/V−/USV− patients (*p* < 0.05, FC = 1.23) (Figure 1). Alterations in SM levels have been previously reported in various forms of hyperlipidemia, including hypercholesterolemia [32,33,34,35,36] and also in CAD [37,38], that is an adverse consequence of FH. As we also observed, even non-genetically confirmed hypercholesterolemic patients (FH/V−/USV−) showed higher SM levels compared to healthy controls [35]. Moreover, we did not observe lower levels of PC and LPC species in hypercholesterolemic patients, as reported elsewhere [34,35], potentially due to the effect of the lipid-lowering therapy, as highlighted in our association analysis (Table 4).

In HoFH patients, an increased proportion of SM in lipoprotein lipids (34.5% of total lipid) compared to HeFH (28.5% of total lipid) has been reported; both groups had higher SM compared to normolipidemic controls (23.1% of total lipid) [36]. Complementing this, the absolute plasma concentration of SM is also significantly and progressively higher in HoFH patients [34]. Furthermore, a lipidomic study [37] has shown that patients with high probability of FH exhibit a distinct sphingolipid profile, including a significant increase in specific long-chain SM, suggesting that these lipid alterations are a specific feature of the disease. In our study, to demonstrate that the observed lipid alteration was specific to SM and not merely a general increase in plasma phospholipids, the SM/(PC + SM) ratio was assessed. The results (Supplementary Material, Figure S4) strengthen the specific dysregulation of SM metabolism [37].

These observations are not limited to humans. In the Watanabe Heritable Hyperlipidaemic rabbit, a well-established animal model of FH caused by a defect in LDLR function and in lipoprotein particles, significantly increased SM levels were reported compared to healthy controls and to another FH animal model where the pathology is driven by ApoB overproduction [39]. The distinct lipid profile in the LDLR-deficient model, characterized by elevated SM, also suggests a specific surface coat abnormality that contributes to the disease phenotype.

While the existing literature associates altered SM levels with FH, their specific profiles in the context of well-defined genetic etiologies (HeFH vs. FH/V−/USV−) have not been explored in humans thus far. Our study confirmed that SM levels are higher in hypercholesterolemic patients compared to normolipidemic controls and also allowed us to discriminate patients with and without pathogenic variants. In our study, SM (d38:4), SM (d44:4), SM (d43:2), and SM (d32:0) drove the intergroup separation in PLS-DA analysis (Figure 1A; Table 3) and showed good discriminating power in the ROC curve analysis.

Sphingolipids, including both the Cer and SM classes, play a structural role in the cell membrane and act as signalling molecules. In particular, the degradation of SM regulates cholesterol biosynthesis [40], and have recently raised interest as potential therapeutic targets in CAD [41]. Sphingolipids have been shown to influence LDL function in tissues, with LDL-associated SM playing a role in their aggregation and macrophage accumulation as well as in modulating LDL kinetics. This process is mediated by sphingomyelinase activity within coronary plaques, which converts SM into Cer [41]. Moreover, a primary impairment in SM uptake or a structural abnormality of the cell membrane has been proposed to explain higher SM levels in HeFH patients’ derived fibroblasts even when LDL-C is similar to healthy controls [42].

Taken together, these data suggest that in HeFH patients, the presence of a pathogenic variant may not only lead to an increase in LDL-C levels but also to a remodelling of the particle’s lipid composition. We propose that this process leads to a selective enrichment of SM on the lipoprotein surface, rendering them more susceptible to aggregation and oxidation even in the absence of a significant increase in LDL-C [41]. These findings can explain the increased susceptibility to CVD in the presence of an FH pathogenic variant [13]. Furthermore, decreased surface total phosphatidylcholine content with raised SM and saturated fatty acid contents have been reported in HDL particles isolated from FH patients [43], highlighting an altered composition of this lipoprotein class and suggesting its dysfunction as well as altered HDL-C levels [44].

Our results not only confirm previous data but also highlight a difference related to the presence of a pathogenic variant among clinically suspected FH. An additional strength of these results is due to the exclusion of patients with USV among the group of FH patients without pathogenic variants. It was observed that FH patients with USV showed an intermediate phenotype between HeFH and patients without pathogenic variant [45]. These data emphasize the importance of sphingolipid metabolism in the presence of FH pathogenic variants, suggesting a potential role in the increased susceptibility to atherosclerosis observed in FH patients and highlighting SM as potential therapeutic targets to reduce CVD risk.

The current literature broadly compares hypercholesterolemic patients to healthy controls [46,47], often without distinguishing between genetically confirmed FH and clinically diagnosed FH. While another study [33] investigated the lipidomic features in patients classified by different probabilities of FH (definite, probable, and possible FH according to Dutch Lipid Clinic Network criteria), our study provides a crucial distinction by directly comparing patients with genetically confirmed HeFH to those with a clinical diagnosis of FH but without an identified pathogenic variant or even a potentially pathogenic variant (FH/V−/USV−). This genetic stratification, which is a key novel aspect of our work, allows for a more precise understanding of how specific genetic defects influence the sphingolipid profile, distinguishing it from broader clinical hypercholesterolemia. To the best of our knowledge, only one study has compared the lipidomic signature of FH patients with healthy controls divided into low and high LDL-C levels, identifying in this latter group a profile similar to FH patients [48].

We acknowledge that limitations are related to the low number of samples, which resulted in an underpowered study. However, the patient number was due to the retrospective nature of the study design, which aimed at the evaluation of patients with a strict genetic characterization. For these reasons, the inclusion of patients carrying variants with an undetermined role in FH pathogenicity was avoided. Furthermore, data about metabolic-related diseases such as diabetes, obesity, and levels of inflammatory markers such as C-reactive protein were not available. Nevertheless, despite some missing data from the patients involved, the key characteristics of the study are homogeneous, as no strong outliers were detected within the same lipid classes.

## 4. Materials and Methods

### 4.1. Study Population and Genetic Analysis

Adult patients with clinical suspicion of FH were enrolled based on LDL-C levels ≥ 140 mg/dL and a family history of hypercholesterolemia and/or of premature coronary artery disease (<55 years in men or <60 years in women). We used a lower threshold because we observed mild hypercholesterolemia among genetically diagnosed patients [44,49]. None of the patients had suspected other lipid-related diseases, such as fatty liver disease

Twenty-two healthy controls were enrolled based on a normolipidemic profile and the absence of a current lipid-lowering therapy.

The recruitment was performed at the Dipartimento di Medicina Clinica e Chirurgia of Università degli Studi di Napoli Federico II. Genetic analysis of patients was performed by Next Generation Sequencing (NGS) using the Devyser FH v2 kit (Devyser, Årsta, Sweden) as previously described [50]. All samples were EDTA-plasma collected from fasting subjects. Briefly, we sequenced the exon-intron junctions of *LDLR*, *APOB*, *PCSK9*, *LDLRAP1*, and *APOE* genes by 2 × 150 base pair sequencing with Kit v2 Micro on the MiSeq platform (Illumina, San Diego, CA, USA) and data analysis was performed by the Amplicon Suite software version 3.5.1 (SmartSeq-Devyser, Årsta, Sweden), which also allowed us to evaluate copy number variants (CNVs) of the *LDLR* gene. The most recent recommendation for pathogenicity evaluation of variants in FH-related genes was followed [51] for variants in the *APOB* and *PCSK9* genes and [52] for variants in the *LDLR* gene. To compare patients with and without pathogenic variants, we considered only HeFH patients (n = 20) to obtain a homogeneous group, excluding the homozygous ones that showed a dramatic lipid profile and could have led to false estimation of the results. For homogeneity, only HeFH patients with pathogenic variants in *LDLR* were included. Furthermore, as to patients without pathogenic variants, we included only patients who did not carry any USV (FH/V−/USV−; n = 19), as these are rare variants for which a pathogenic role cannot be excluded. The biochemical data reported in Table 1 (total cholesterol, LDL-C, HDL-C, non-HDL-C, ApoB, and TG) were obtained by the enzymatic method.

### 4.2. Lipid Extraction and Analysis

For lipid quantification, plasma samples were spiked with a commercial lipid standard mixture (SPLASH™ LIPIDOMIX™ Mass Spec, Avanti Research, Merck, Milan, Italy) which included (final concentration in samples reported in parentheses) 15:0–18:1 (d7) PC (16.07 µg/mL):15:0–18:1 (d7) PE (0.57 µg/mL), 15:0–18:1 (d7) PG (2.91 µg/mL), 18:1 (d7) LysoPC (2.55 µg/mL), 18:1 (d7) LysoPE (0.53 µg/mL), 18:1 (d7) ChE (35.61 µg/mL), 15:0–18:1 (d7) DG (0.94 µg/mL), 15:0–18:1(d7)–15:0 TG (5.73 µg/mL), and 18:1 (d9) SM (3.09 µg/mL). Additionally, C17 Ceramide (d18:1/17:0) (0.25 µg/mL) (Avanti Research, Merck, Milan, Italy) was incorporated.

Lipid extraction was performed according to a modified Matyash method [53,54]. Briefly, 20 µL of plasma spiked with the standard mixture were thoroughly mixed with 300 µL of methanol. Subsequently, 1 mL of methyl tert-butyl ether (MTBE) was added. The mixture was sonicated for 1 min (in ice bath) and then gently shaken at 10 °C for 1 h. Phase separation was achieved by adding 250 µL of milliQ water and shaking for 10 min. Following centrifugation (10 min, 10 °C, 10,000× *g*), the upper organic phase was carefully transferred to a pre-weighed glass vial. The remaining lower phase, including the pellet, underwent a secondary extraction with 300 µL of MTBE by sonication and gentle shaking at 10 °C for 1 h. After centrifugation, the pooled organic phases were dried under nitrogen stream. Dried lipid extracts were stored at −20 °C until LC-MS analysis.

For lipidome LC-MS analyses, each sample was reconstituted in 100 µL of acetonitrile/isopropanol (70:30) and analyzed by UHPLC-MS/MS. Chromatographic separation was performed at 40 °C on Infinity 1290 UHPLC System (Agilent Technologies, Santa Clara, CA, USA) equipped with an Acquity UPLC CSH C18 column (1.7 µm, 100 mm × 2.1 mm, Waters, Milford, MA, USA) by applying a gradient elution program using eluent A (water/acetonitrile 60:40, 10 mM ammonium formate, 0.1% formic acid) and eluent B (isopropanol/acetonitrile 90:10, 2 mM ammonium formate, 0.1% formic acid) as follows: 0–2.5 min, 15% to 50% (B); 2.5–10.5 min, 50% to 80% (B); 10.5–11.0 min, 80% to 99% (B); 11.0–12.0 min holding to 99% (B); then a return to initial condition in 0.1 min and equilibration for 3 min. The column temperature was set to 40 °C and the flow rate was 0.3 mL/min. Mass spectrometry analyses were conducted on a Q-Exactive Hybrid Quadrupole-Orbitrap mass spectrometer (Thermo Scientific, San Jose, CA, USA) in both positive and negative polarity modes over a mass range of *m*/*z* 150–1800.

### 4.3. Lipid Identification

Raw UHPLC-Q Exactive MS/MS data were processed using LipidSearch Software (version 4.1.30, Thermo Scientific, San Jose, CA, USA). Precursor ion tolerance was set to 5 ppm and product ion tolerance to 10 ppm. An m-score threshold of 5 was applied. Identified lipid lists were aligned and compared based on their lipid class and species levels, utilizing a retention time tolerance of ±0.25 min. A main grade of A, B, or C was assigned for all lipid classes as previously described [54]. Primary adduct ions for specific lipid classes were defined as follows: H^+^ for PC, PE, LysoPC, and LysoPE; NH_4_^+^ for DG and TG. Ceramide species were measured as HCOO^-^ adducts, while PI, PS, and PG were monitored as deprotonated ions. After peak alignment, the software yielded an output of 694 lipid ions, divided into the classes reported above (Supplementary Material Figure S1). Regiochemistry was not assigned. All lipid species were manually double-checked and quantified by normalizing to the respective internal standard for each class (Supplementary Material Table S1).

### 4.4. Metabolite Extraction and NMR Spectral Acquisition

The plasma samples (400 µL) were stored at −80 °C, thawed, diluted 1:1 *v*/*v* with Phosphate Buffered Saline 1× (PBS, pH 7.4), and filtered using Amicon Ultra-2 mL 10,000 MWCO centrifugal filters (Merck, Milan, Italy) at 10,000 rpm for 12 min to physically remove interfering proteins and extract the low molecular weight molecules (metabolites). Before plasma filtration, each centrifugal filter was pre-rinsed several times using ∼2 mL distilled water and centrifuged at 10,000 rpm for 10 min to remove residual amounts of glycerol from the membrane filters, thereby avoiding interference with surrounding NMR signals and/or erroneous glycerol quantification. Each sample filtrate (450 µL) was mixed with 180 µL of PBS 1× (pH 7.4) and combined with 70 µL of D_2_O plus TSP (trimethylsilyl-2,2,3,3-tetradeuteropropionic acid) 1 mM, which was used as a chemical shift reference for ^1^H spectra and transferred into 5-mm NMR tubes for NMR analysis.

NMR spectra were acquired on a Bruker Avance III 600 MHz spectrometer (BrukerBioSpin GmbH, Rheinstetten, Germany) equipped with a TCI CryoProbe fitted with a gradient along the Z-axis at a probe temperature of 300 K (27 °C). In particular, 1D spectra were acquired, including water suppression with excitation sculpting sequence [55] together with homo- and heteronuclear 2D experiments (^1^H–^1^H clean total-correlation spectroscopy TOCSY, heteronuclear single quantum coherence HSQC ^1^H–^13^C HSQC, and heteronuclear multiple bound correlation HMBC ^1^H–^13^C), providing monodimensional metabolic profiles and homonuclear and heteronuclear spectra for metabolite identification. Metabolite assignments were achieved by comparing signal chemical shifts with the literature [56] and an online database [57].

### 4.5. NMR Data Analysis

Plasma ^1^H-NMR spectra were processed and normalized to the total spectrum area to avoid possible dilution effects on the signals. Water resonance together with EDTA signals were removed from each spectral range (8.60 to 0.60 ppm) and automatically binned into 330 integrals of 0.02 ppm each using the AMIX 3.9.15 software package (Bruker Biospin GmbH, Rheinstetten, Germany), which was arranged as a data matrix.

### 4.6. Statistical Analysis

Univariate statistical analyses were conducted using GraphPad Prism (version 10, GraphPad Software, San Diego, CA, USA). Continuous variables with a non-normal distribution were presented as median with interquartile range (IQR) and compared using the non-parametric *t*-test (Mann–Whitney test). For multiple group comparisons, non-parametric ANOVA tests (Kruskal–Wallis test) were performed. Comparison of clinical characteristics between two groups of participants was performed by the Kruskal–Wallis test for continuous variables and Chi-square (χ2) test for categorical variables. Correlations between selected metabolites and fasting lipid profiles were assessed using Spearman’s rank correlation coefficient. A *p*-value < 0.05 was considered statistically significant.

The between-subject data were then Pareto-scaled and lipidome differences between sample were maximized using Partial Least Squares Discriminant Analysis (PLS-DA) and Ortho(O)PLS-DA, and key variables contributing to the classification were identified according to their variable importance in projection value (VIP), using MetaboAnalyst Version 6.0. Variables with a VIP > 1, a *p* < 0.05, and a |fold-change| (FC) of >1.2 were screened out and identified as potential biomarkers.

Receiver Operating Characteristic (ROC) curves were generated using GraphPad Prism (version 8, GraphPad Software, San Diego, CA, USA) to assess the discriminatory power of individual metabolites. Lipids with an Area Under the Curve (AUC) > 0.6 were considered to have at least moderate discriminative ability. Multivariable linear regression analysis was used to assess the association between lipid concentrations and genotype, adjusting for lipid-lowering therapy, age, and sex. Only metabolites/lipids with a VIP score > 1 in the PLS-DA model and a *p*-value < 0.05 in univariate analysis were included in the regression models in order to focus on the most relevant metabolic features discriminating between hypercholesterolemic patients.

## 5. Conclusions

In this study, we provide evidence that plasma lipidomics can capture metabolic differences between patients with FH carrying pathogenic variants in heterozygosis in *LDLR* and those without identifiable genetic variants, even those of uncertain significance, reinforcing the role of sphingolipid metabolism in hypercholesterolemia and potentially in the related cardiovascular complications. Despite similar clinical and lipid profiles, distinct lipid species—particularly within SM class—drive the group separation, were associated with the genetic status, and showed strong correlations with traditional lipid markers, including LDL-C, total cholesterol, and non-HDL-C. These findings suggest on the one hand that lipidomics may offer additional insights into the biochemical variability of FH beyond conventional blood parameters; on the other hand, the results support the measurement of total SM as a cue of increased cardiovascular risk associated with pathogenic variant carriage in FH. Because SM quantification can be easily performed using commercially available kits [37,58,59], this approach could be translated into routine laboratory practice. SM biosynthetic pathway and/or SMase inhibition have already been proposed as feasible pharmacological targets in combination with other therapeutic agents, including PCSK9i, in patients where the level of this class of lipids is significantly altered [60,61,62,63]. Although robust diagnostic thresholds for SM must be assessed to aid in risk stratification, our study proposes the identification of patients that may particularly benefit of these personalized strategies. However, we acknowledge that the limited number of subjects enrolled for the present study requires further investigation in larger and independent cohorts, as well as evaluating co-morbidities that include other metabolic disorders, in order to validate the observed lipid signatures and assess their potential as biomarkers for increased CVD risk in FH.

## Figures and Tables

**Table 1:** Baseline characteristics of the study population, including patients with Heterozygous FH (HeFH), FH patients without pathogenic or uncertain significance variants (FH/V−/USV−), and healthy individuals (CTR).

Characteristic	Controls (CTR)	Heterozygotes for a Pathogenic Variant (HeFH)	Without Pathogenic/ Uncertain Significance Variants (FH/V−/USV−)	HeFH vs. CTR	FH/V−/USV− vs. CTR	HeFH vs. FH/V−/USV−
	(n = 22)	(n = 20)	(n = 19)	Adjusted *p*-Value		
Demographics						
Age (mean years ± SD)	46.6 ± 12.0	46.1 ± 14.2	58.5 ± 13.1	>0.9999	**0.0268**	**0.0249**
Sex (% female)	72.7%	65.0%	63.2%	0.7828		
Biochemical Parameters						
Total cholesterol (mg/dL)	160.4 ± 15.8	203.2 ± 50.2	183.2 ± 63.5	**0.0213**	>0.9999	0.2473
LDL-C (mg/dL)	94.5 ± 14.3	131.2 ± 52.4	104.8 ± 61.3	**0.0500**	0.9852	0.0800
HDL-C (mg/dL)	56.3 ± 12.0	49.0 ± 10.4	50.5 ± 15.6	0.1802	0.4096	>0.9999
Non-HDL-C (mg/dL)	104.1 ± 16.5	154.1 ± 50.5	132.7 ± 59.6	**0.0018**	0.5278	0.1392
Triglycerides (mg/dL)	73.6 ± 24.4	106.3 ± 60.0	128.5 ± 90.0	0.1260	**0.0064**	0.8910
ApoB (mg/dL)	69.7 ± 10.7	103.8 ± 37.5	90.6 ± 38.1	**0.0003**	0.1482	0.1831
Lipid Lowering Therapy *^,#^						
Low/moderate-intensity	\	6	10	0.1005		
High-intensity	\	13	7			
PCSK9 inhibitors	\	0	2			

* Only for FH patients. Ezetimibe and bempedoic acid were considered low-intensity treatments. Statins were classified based on their lipid-lowering potency and dosage: Atorvastatin (10–20 mg), Rosuvastatin (5–10 mg), and Simvastatin (40 mg) were categorized as low-to-moderate intensity treatments, while Atorvastatin (40–80 mg) and Rosuvastatin (20–40 mg) were classified as high-intensity treatments. # One HeFH patient not in therapy because of lactation status.

**Table 2:** Normalized bin values for discriminant metabolites presented as median and IQR (Q1–Q3) in arbitrary units for each of the three study groups. Adjusted *p*-values for multiple comparisons are provided for the following group comparisons: patients with Heterozygous Familial Hypercholesterolemia (HeFH) versus healthy individuals (CTR), patients without Pathogenic/Uncertain Significance variants (FH/V−/USV−) versus CTR, and HeFH versus FH/V−/USV−.

Metabolite	CTR	HeFH	FH/V−/USV−	Group Comparison p_adj_ *		
	Median (IQR)	Median (IQR)	Median (IQR)	HeFH vs. CTR	FH/V−/USV− vs. CTR	HeFH vs. FH/V−/USV−
proline	4.5 × 10^−4^	6.7 × 10^−4^	7.8 × 10^−4^	0.0010	<0.0001	0.587
	(3.7 × 10^−4^–5.1 × 10^−4^)	(6.0 × 10^−4^–7.5 × 10^−4^)	(6.3 × 10^−4^–8.7 × 10^−4^)			
glutamate	4.3 × 10^−4^	7.8 × 10^−4^	8.4 × 10^−4^	<0.0001	<0.0001	1
	(3.4 × 10^−4^–4.7 × 10^−4^)	(7.0 × 10^−4^–8.7 × 10^−4^)	(7.7 × 10^−4^–9.0 × 10^−4^)			
threonine	9.8 × 10^−4^	0.002	0.003	<0.0001	<0.0001	1
	(8.9 × 10^−4^–11.7 × 10^−4^)	(0.002–0.003)	(0.002–0.003)			
3-hydroxybutyrate	10.3 × 10^−4^	0.001	0.002	0.0124	0.0001	0.724
	(9.3 × 10^−4^–14.9 × 10^−4^)	(0.001–0.002)	(0.001–0.002)			
2-hydroxybutyrate	2.1 × 10^−4^	2.7 × 10^−4^	2.7 × 10^−4^	0.0331	0.0015	1
	(1.6 × 10^−4^–2.6 × 10^−4^)	(2.1 × 10^−4^–3.7 × 10^−4^)	(2.4 × 10^−4^–3.4 × 10^−4^)			
isoleucine	4.6 × 10^−4^	5.6 × 10^−4^	6.2 × 10^−4^	0.0013	<0.0001	1
	(4.3 × 10^−4^–5.2 × 10^−4^)	(4.9 × 10^−4^–7.0 × 10^−4^)	(5.5 × 10^−4^–6.7 × 10^−4^)			
lysine	4.9 × 10^−4^	4.7 × 10^−4^	7.3 × 10^−4^	<0.0001	<0.0001	1
	(4.2 × 10^−4^–5.5 × 10^−4^)	(6.0 × 10^−4^–8.1 × 10^−4^)	(6.4 × 10^−4^–8.0 × 10^−4^)			
lactate	0.019	0.025	0.039	0.323	<0.0001	0.118
	(0.017–0.024)	(0.021–0.037)	(0.027–0.052)			
glutamine	6.1 × 10^−4^	3.4 × 10^−4^	3.8 × 10^−4^	<0.0001	0.0001	0.877
	(5.4 × 10^−4^–6.4 × 10^−4^)	(3.0 × 10^−4^–4.2 × 10^−4^)	(2.5 × 10^−4^–5.2 × 10^−4^)			
acetate	0.0012	8.2 × 10^−4^	0.0010	<0.0001	0.0245	0.291
	(0.0010–0.0012)	(7.0 × 10^−4^–9.9 × 10^−4^)	(0.0007–0.0012)			
tyrosine	9.2 × 10^−4^	6.7 × 10^−4^	6.8 × 10^−4^	<0.0001	<0.0001	1
	(8.8 × 10^−4^–9.9 × 10^−4^)	(5.9 × 10^−4^–7.5 × 10^−4^)	(5.5 × 10^−4^–7.2 × 10^−4^)			
glucose	0.0021	0.0017	0.0021	0.005	1	0.09
	(0.0020–0.0023)	(0.0015–0.0019)	(0.0016–0.0025)			

* Statistical analysis utilized either an ANOVA test followed by Bonferroni correction for normally distributed data, or a Kruskal–Wallis and Dunn’s test with Bonferroni correction for non-normally distributed data.

**Table 3:** Significantly altered lipids with VIP > 1 between HeFH and FH/V−/USV− patients (reported as median (µg/mL) and interquartile range (IQR; Q1–Q3)) (Mann–Whitney test, Sign: *p* < 0.05).

		HeFH		FH/V−/USV−		Adjusted *p*-Value *	FC
Lipid	Class	Median (µg/mL)	IQR	Median (µg/mL)	IQR		
Cer (d18:1/23:0)	Cer	0.73	0.61–0.92	1.02	0.77–1.62	0.0283	−1.41
LPC (20:4)	LPC	5.51	4.82–6.56	4.23	2.96–5.26	0.0243	1.30
PC (18:0/20:4)	PC	102.60	90.64–119.47	80.53	53.50–106.50	0.0168	1.27
PE (18:0p/18:2)	PE	0.38	0.01–1.06	1.17	0.48–1.76	0.0283	−3.09
SM (d32:0)	SM	0.50	0.41–0.78	0.35	0.31–0.49	0.0256	1.44
SM (d38:4)	SM	0.76	0.64–0.93	0.47	0.44–0.65	0.0056	1.63
SM (d43:2)	SM	5.17	4.01–7.88	3.15	1.89–5.00	0.0116	1.64
SM (d44:4)	SM	6.47	3.83–7.52	3.98	2.67–5.75	0.0242	1.62

* Statistical analysis utilized a Mann–Whitney test with Benjamini–Hochberg (FDR) correction for non-normally distributed data.

**Table 4:** Multivariate linear regression analysis examining the association of genotype (HeFH vs. FH/V−/USV−), therapy, sex, and age with the levels of key lipid species identified by PLS-DA. Coefficients (β) and *p*-values are reported for each predictor.

Lipid	Presence of FH Pathogenic Variants (β, *p*-Value)	Lipid-Lowering Therapy (β, *p*-Value)	Sex (β, *p*-Value)	Age (β, *p*-Value)
Cer (d18:1/23:0)	−0.27, *p* = 0.0731	−0.05, *p* = 0.7018	0, *p* = 0.9695	0, *p* = 0.8129
LPC (20:4)	1.11, *p* = 0.0733	**1.45**, *p* = **0.0134**	−0.64, *p* = 0.2509	0.02, *p* = 0.2380
PC (18:0/20:4)	**21.51**, *p* = **0.0193**	**20.18**, *p* = **0.0180**	−6.29, *p* = 0.4382	0.23, *p* = 0.4473
PE (18:0p/18:2)	−0.56, *p* = 0.0584	−0.03, *p* = 0.9058	**0.60**, *p* = **0.0277**	0, *p* = 0.7687
SM (d32:0)	**0.22**, *p* = **0.0084**	−0.10, *p* = 0.1654	0.01, *p* = 0.8882	0, *p* = 0.8075
SM (d38:4)	**0.22**, *p* = **0.0059**	0.05, *p* = 0.5095	−0.08, *p* = 0.2503	0, *p* = 0.7967
SM (d43:2)	**2.99**, *p* = **0.0003**	−1.23, *p* = 0.0788	−0.20, *p* = 0.7711	0.03, *p* = 0.1599
SM (d44:4)	**2.25**, *p* = **0.0118**	−1.22, *p* = 0.1271	−0.24, *p* = 0.7532	0, *p* = 0.8932
SM (total)	**190.13**, *p* = **0.0256**	−104.18, *p* = 0.1754	21.23, *p* = 0.7776	0.03, *p* = 0.9919

## Data Availability

Data are available upon reasonable request to the corresponding author.

## Supplementary Materials

The following supporting information can be downloaded at: https://www.mdpi.com/article/10.3390/ijms262110688/s1.
