# Supplementary material for: Lipidomic Signature of Patients with Familial Hypercholesterolemia Carrying Pathogenic Variants Unveils a Cue of Increased Cardiovascular Risk
Source: Int J Mol Sci. 2025 Nov 3;26(21):10688. doi: 10.3390/ijms262110688 (PMC12609719; doi:10.3390/ijms262110688)
Supplement: Supplementary file 1 [file ijms-26-10688-s001.zip › ijms-3927157-supplementary.pdf]

Supplementary materials

Lipidomic signature of patients with familial hypercholesterolemia carrying pathogenic variants unveils a cue of increased cardiovascular risk

Giulia De Simone[1], Maria Donata Di Taranto[2,3], Debora Paris[1], Martina Ferrandino[2,3], Marco Andolfi[1], Annalaura Iodice[1], Giovanna Cardiero[2,3], Carmine De Luca[4,5], Luigi Junior Valletta[4,5], Ilenia Lorenza Calcaterra[4,5], Gabriella Iannuzzo[4,5], Matteo Nicola Dario Di Minno[4,5], Giuliana Fortunato[2,3], Adele Cutignano[1,*]

[1] Consiglio Nazionale delle Ricerche (CNR), Istituto di Chimica Biomolecolare (ICB), via Campi Flegrei 34, 80078 Pozzuoli, Italy
[2] Dipartimento di Medicina Molecolare e Biotecnologie Mediche, Università degli Studi di Napoli Federico II, via Pansini 5, 80131 Napoli, Italy
[3] CEINGE Biotecnologie Avanzate Franco Salvatore, Via Gaetano Salvatore, 486, 80145 Napoli, Italy
[4] Dipartimento di Medicina Clinica e Chirurgia, Università degli Studi di Napoli Federico II, via Pansini 5, 80131 Napoli, Italy
[5] Centro di riferimento regionale di Diagnosi e terapia delle dislipidemie nell'adulto, AOU Federico II, via Pansini 5, 80131 Napoli, Italy

Contents:

Figure S1: Treemap chart of the LipidSearch software output of lipid species in plasma samples processed by UHPLC-HRESIMS/MS.

Figure S2: A) OPLS-DA scores plot showing plasma samples projected onto the predictive components t[1] and t[2]. B) Loadings plot reporting the NMR bins (chemical shift) responsible for data clusters in the scores plot. The color of the variables is encoded with the value of the correlation (pcorr) between the pq loadings and the bin values.

Figure S3: Faceted heatmap displaying Spearman correlations between significantly altered SM and clinical data in CTR group

Figure S4: A) Box plot representing the SM/SM+PC ratio in HeFH, FH/V−/USV− and CTR (Kruskal-Wallis test; * $p < 0.05$); B) Correlation between SM/SM+PC ratio and SM levels (µg/mL), in HeFH (green), FH/V−/USV− (blue), and CTR (red)

Table S1: Lipid species quantified in plasma samples of HeFH, FH/V−/USV− and CTR groups. Values are reported as µg/mL.

Table S2: Lipid classes reported as Median [Interquartile Range (Q1-Q3)] in the three groups examined. Fold change (FC) values are reported for the comparisons between patients with Heterozygous Familial Hypercholesterolemia (HeFH) versus Controls (CTR), patients without Pathogenic / Uncertain Significance variants (FH/V−/USV−) versus CTR, and HeFH versus FH/V−/USV−. (***Bolded FC values*** indicate classes with statistically significant differences ($p < 0.05$) as determined by the Kruskal-Wallis test with FDR correction)

Table S3: Correlation results (Spearman R and p-value) between lipid classes and clinical data in the overall cohorts, control (CTR), HeFH, and FH/V−/USV−.

Table S4: Area Under the Curve (AUC) and 95% Confidence interval of the significant Sphingomyelin species

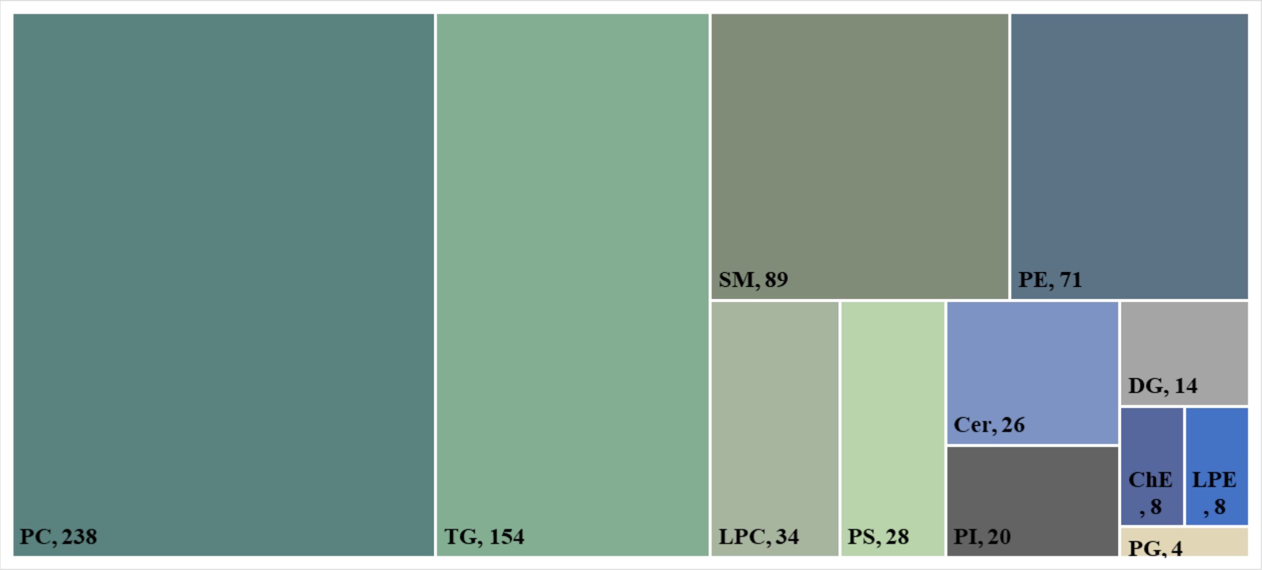

Figure S1: Treemap chart of the LipidSearch software output of lipid species in plasma samples processed by UHPLC-HRESIMS/MS.

.

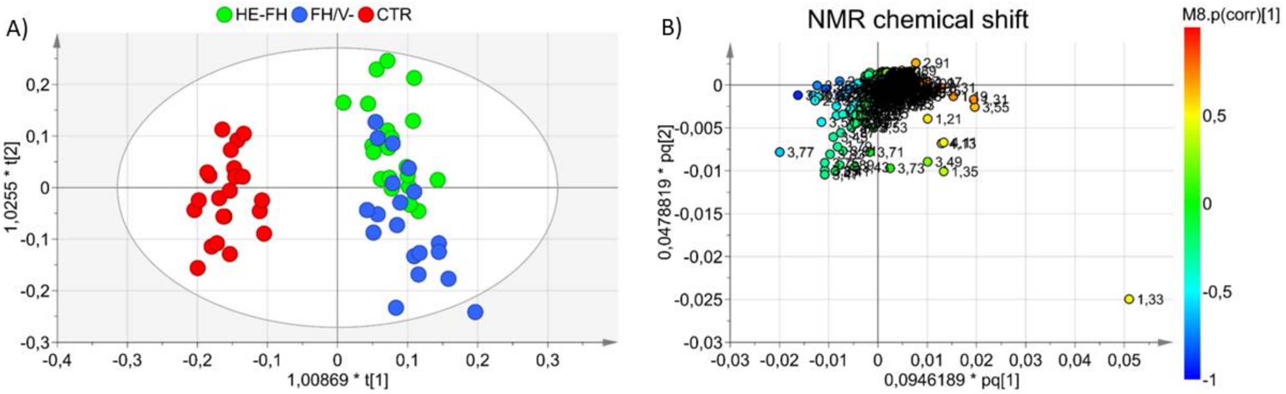

Figure S2: A) OPLS-DA scores plot showing plasma samples projected onto the predictive components t[1] and t[2]. B) Loadings plot reporting the NMR bins (chemical shift) responsible for data clusters in the scores plot. The color of the variables is encoded with the value of the correlation (pcorr) between the pq loadings and the bin values.

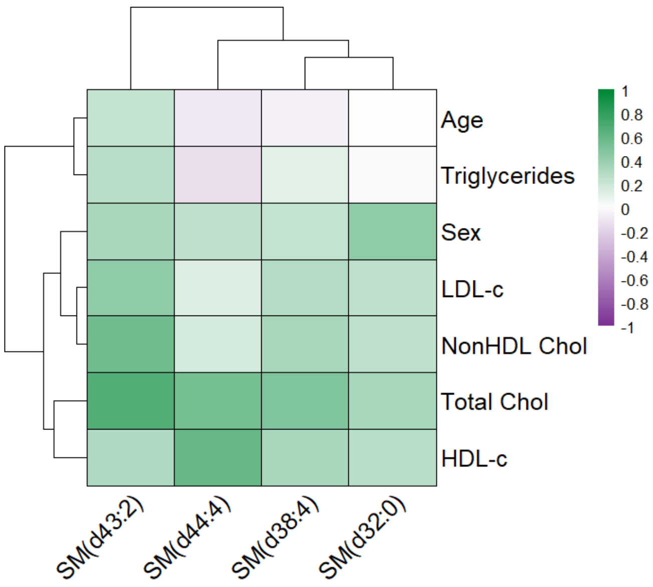

Figure S3: Faceted heatmap displaying Spearman correlations between significantly altered SM and clinical data in CTR group

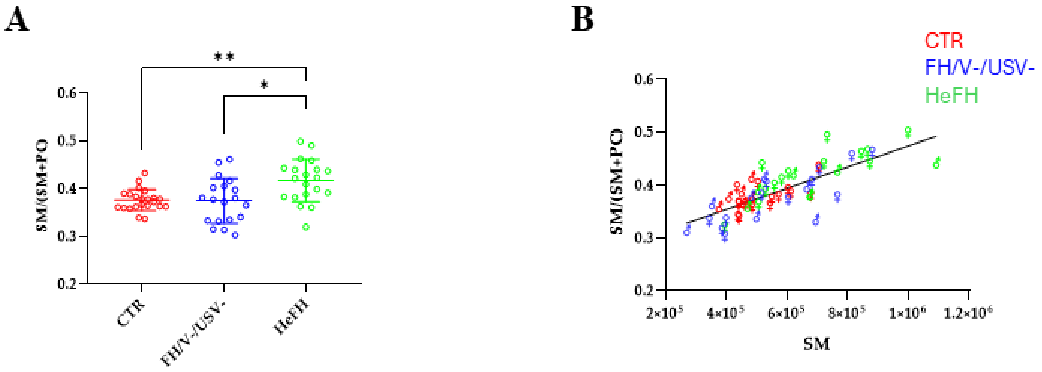

Figure S4: A) Box plot representing the SM/SM+PC ratio in HeFH, FH/V−/USV− and CTR (Kruskal-Wallis test; * $p < 0.05$); B) Correlation between SM/SM+PC ratio and SM levels (μg/mL), in HeFH (green), FH/V−/USV− (blue), and CTR (red)

Table S1: Lipid species quantified in plasma samples of HeFH, FH/V−/USV− and controls (CTR) groups. Values are reported as μg/mL.

Lipid species	Class	FA Group Key	MainIon	Mass	Formula	Rt	Median CTR	SEM CTR	Median HeFH	SEM HeFH	Median FH/V-/USV-	SEM FH/V-/USV-
Cer(d16:1/22:0)	Cer	d38:1	+H	593.5747	C38 H75 O3 N1	11.118	0.10	0.01	0.16	0.02	0.15	0.02
Cer(d18:0/22:0)	Cer	d40:0	+H	623.6216	C40 H81 O3 N1	11.893	0.15	0.01	0.25	0.02	0.19	0.03
Cer(d18:0/23:0)	Cer	d41:0	+H	637.6373	C41 H83 O3 N1	12.007	0.10	0.01	0.17	0.02	0.16	0.02
Cer(d18:0/24:0)	Cer	d42:0	+H	651.6529	C42 H85 O3 N1	12.177	0.37	0.02	0.96	0.10	0.81	0.09
Cer(d18:1/22:0)	Cer	d40:1	+H	621.606	C40 H79 O3 N1	11.827	0.61	0.05	1.05	0.11	1.03	0.12
Cer(d18:1/23:0)	Cer	d41:1	+H	635.6216	C41 H81 O3 N1	12.067	0.61	0.05	0.73	0.08	1.02	0.09
Cer(d18:1/24:0)	Cer	d42:1	+H	649.6373	C42 H83 O3 N1	12.202	2.51	0.15	3.54	0.32	4.25	0.36
Cer(d18:1/24:1)	Cer	d42:2	+H	647.6216	C42 H81 O3 N1	11.778	1.17	0.07	1.29	0.10	1.50	0.15
Cer(d18:1/25:0)	Cer	d43:1	+H	663.6529	C43 H85 O3 N1	12.274	0.49	0.04	0.66	0.07	0.69	0.07
Cer(d18:2/24:1)	Cer	d42:3	+H	645.606	C42 H79 O3 N1	11.125	0.13	0.01	0.18	0.02	0.24	0.03
ChE(16:0)	ChE	16:0	NH4	624.5845	C43 H76 O2	12.9	16.10	0.37	37.71	3.42	47.77	3.46
ChE(18:1)	ChE	18:1	NH4	650.6002	C45 H78 O2	12.954	838.79	30.85	961.60	83.13	1116.95	114.31
ChE(18:2)	ChE	18:2	NH4	648.5845	C45 H76 O2	12.839	3659.15	117.28	3949.98	331.81	4130.76	446.08
ChE(18:3)	ChE	18:3	NH4	646.5689	C45 H74 O2	12.653	43.62	3.35	126.26	20.10	143.37	18.08
ChE(20:4)	ChE	20:4	NH4	672.5845	C47 H76 O2	12.764	899.65	53.15	2194.85	347.34	1921.11	216.21
ChE(20:5)	ChE	20:5	NH4	670.5689	C47 H74 O2	12.136	28.56	4.85	252.99	40.62	296.92	88.02
ChE(22:6)	ChE	22:6	NH4	696.5845	C49 H76 O2	12.677	168.42	14.66	358.22	40.18	307.98	52.67
DG(16:0/18:1)	DG	34:1	NH4	594.5223	C37 H70 O5	10.828	1.49	0.18	2.50	0.50	3.06	0.94
DG(16:1/18:1)	DG	34:2	NH4	594.5223	C37 H70 O5	10.544	1.43	0.19	1.82	0.43	2.88	0.57
DG(18:0/18:1)	DG	36:1	NH4	622.5536	C39 H74 O5	11.558	0.81	0.05	0.58	0.13	0.59	0.20
DG(18:1/18:1)	DG	36:2	NH4	620.538	C39 H72 O5	10.878	6.35	0.78	5.68	1.09	9.30	1.23
DG(18:1/18:2)	DG	36:3	NH4	618.5223	C39 H70 O5	10.242	7.56	0.95	5.05	0.98	6.70	0.96
DG(18:1/20:4)	DG	38:5	NH4	642.5223	C41 H70 O5	9.99	1.61	0.13	1.62	0.27	1.17	0.24
DG(18:2/18:2)	DG	36:4	NH4	616.5067	C39 H68 O5	9.567	2.77	0.34	1.62	0.21	1.79	0.26
LPC(14:0)	LPC	14:0	+H	467.3012	C22 H46 O7 N1 P1	2.445	0.38	0.02	0.41	0.04	0.43	0.06
LPC(15:0)	LPC	15:0	+H	481.3168	C23 H48 O7 N1 P1	2.929	0.30	0.01	0.38	0.03	0.40	0.04
LPC(16:0)	LPC	16:0	+H	495.3325	C24 H50 O7 N1 P1	3.243	30.59	1.08	41.39	5.17	4.95	5.52
LPC(16:0e)	LPC	16:0e	+H	481.3532	C24 H52 O6 N1 P1	3.749	0.24	0.01	0.37	0.03	0.34	0.03

LPC(16:0p)	LPC	16:0p	+H	479.3376	C24 H50 O6 N1 P1	3.687	0.28	0.02	0.42	0.03	0.37	0.02
LPC(16:1)	LPC	16:1	+H	493.3168	C24 H48 O7 N1 P1	2.674	0.75	0.04	1.00	0.06	1.07	0.08
LPC(17:0)	LPC	17:0	+H	509.3481	C25 H52 O7 N1 P1	3.802	0.65	0.03	0.89	0.08	0.83	0.09
LPC(17:1)	LPC	17:1	+H	507.3325	C25 H50 O7 N1 P1	3.144	0.08	0.00	0.10	0.01	0.10	0.01
LPC(18:0)	LPC	18:0	+H	523.3638	C26 H54 O7 N1 P1	4.155	11.28	0.54	20.12	1.30	19.76	1.44
LPC(18:0p)	LPC	18:0p	+H	507.3689	C26 H54 O6 N1 P1	3.867	0.19	0.01	0.31	0.02	0.30	0.02
LPC(18:1)	LPC	18:1	+H	521.3481	C26 H52 O7 N1 P1	3.538	9.45	0.36	14.60	0.82	14.26	0.91
LPC(18:2)	LPC	18:2	+H	519.3325	C26 H50 O7 N1 P1	2.928	13.34	0.93	13.59	0.93	13.86	0.75
LPC(18:3)	LPC	18:3	+H	517.3168	C26 H48 O7 N1 P1	2.408	0.13	0.01	0.12	0.02	0.15	0.02
LPC(20:3)	LPC	20:3	+H	545.3481	C28 H52 O7 N1 P1	3.219	1.04	0.05	1.28	0.10	1.19	0.10
LPC(20:4)	LPC	20:4	+H	543.3325	C28 H50 O7 N1 P1	2.851	2.71	0.13	5.51	0.32	4.23	0.45
LPC(20:5)	LPC	20:5	+H	541.3168	C28 H48 O7 N1 P1	2.326	1.18	0.08	0.47	0.05	0.42	0.03
LPC(22:4)	LPC	22:4	+H	571.3638	C30 H54 O7 N1 P1	3.385	0.05	0.00	0.13	0.02	0.10	0.01
LPC(22:5)	LPC	22:5	+H	569.3481	C30 H52 O7 N1 P1	2.994	0.17	0.01	0.18	0.03	0.17	0.02
LPC(22:6)	LPC	22:6	+H	567.3325	C30 H50 O7 N1 P1	2.732	0.65	0.05	0.86	0.06	0.83	0.09
LPC(24:0)	LPC	24:0	+H	607.4577	C32 H66 O7 N1 P1	7.243	0.01	0.00	0.07	0.00	0.06	0.00
PC(16:0/14:0)	PC	30:0	+H	705.5309	C38 H76 O8 N1 P1	8.295	1.51	0.11	2.37	0.26	2.24	0.45
PC(16:1/14:0)	PC	30:1	+H	703.5152	C38 H74 O8 N1 P1	7.422	0.21	0.02	0.29	0.04	0.30	0.06
PC(15:0/16:0)	PC	31:0	+H	719.5465	C39 H78 O8 N1 P1	8.474	0.41	0.02	0.41	0.04	0.47	0.05
PC(16:0/16:0)	PC	32:0	+H	733.5622	C40 H80 O8 N1 P1	9.21	7.57	0.34	9.77	0.61	9.28	0.78
PC(14:0/18:2)	PC	32:2	+H	729.5309	C40 H76 O8 N1 P1	7.633	2.97	0.22	2.60	0.25	3.22	0.52
PC(16:0/17:0)	PC	33:0	+H	747.5778	C41 H82 O8 N1 P1	9.519	0.49	0.03	0.55	0.06	0.77	0.07
PC(15:0/18:1)	PC	33:1	+H	745.5622	C41 H80 O8 N1 P1	8.849	2.15	0.12	2.75	0.19	3.18	0.30
PC(15:0/18:2)	PC	33:2	+H	743.5465	C41 H78 O8 N1 P1	8.108	2.48	0.16	2.19	0.17	2.15	0.38
PC(18:0/16:0)	PC	34:0	+H	761.5935	C42 H84 O8 N1 P1	10.062	2.36	0.08	3.35	0.24	2.94	0.24
PC(16:0/18:3)	PC	34:3	+H	755.5465	C42 H78 O8 N1 P1	9.207	0.59	0.02	0.34	0.02	0.36	0.03
PC(14:0/20:4)	PC	34:4	+H	753.5309	C42 H76 O8 N1 P1	7.46	1.00	0.09	1.81	0.20	1.66	0.17
PC(14:0/20:5)	PC	34:5	+H	751.5152	C42 H74 O8 N1 P1	7.628	0.04	0.01	0.05	0.01	0.07	0.03
PC(17:0/18:1)	PC	35:1	+H	773.5935	C43 H84 O8 N1 P1	9.689	3.98	0.19	4.62	0.33	4.56	0.34
PC(17:0/18:2)	PC	35:2	+H	771.5778	C43 H82 O8 N1 P1	8.887	8.36	0.38	6.23	0.68	4.42	1.01
PC(17:1/18:2)	PC	35:3	+H	769.5622	C43 H80 O8 N1 P1	8.26	1.23	0.06	0.90	0.10	1.24	0.14
PC(15:0/20:4)	PC	35:4	+H	767.5465	C43 H78 O8 N1 P1	7.942	1.06	0.11	1.51	0.11	1.36	0.15

PC(15:0/20:5)	PC	35:4	+H	765.5309	C43 H76 O8 N1 P1	7.291	0.04	0.01	0.05	0.01	0.07	0.03
PC(18:0/18:1)	PC	36:1	+H	787.6091	C44 H86 O8 N1 P1	10.108	35.09	1.35	45.97	2.97	54.74	3.96
PC(18:1/18:1)	PC	36:2	+H	785.5935	C44 H84 O8 N1 P1	9.462	155.26	3.50	157.98	7.76	180.46	12.58
PC(18:1/18:2)	PC	36:3	+H	783.5778	C44 H82 O8 N1 P1	8.757	100.02	3.64	104.34	7.21	117.69	8.83
PC(16:0/20:4)	PC	36:4	+H	781.5622	C44 H80 O8 N1 P1	8.409	89.85	3.46	170.05	8.47	141.49	9.60
PC(16:0/20:5)	PC	36:5	+H	779.5465	C44 H78 O8 N1 P1	7.765	12.50	1.08	13.27	2.66	12.37	5.54
PC(14:0/22:6)	PC	36:6	+H	777.5309	C44 H76 O8 N1 P1	7.183	0.40	0.04	0.36	0.04	0.41	0.07
PC(19:0/18:2)	PC	37:2	+H	799.6091	C45 H86 O8 N1 P1	9.769	1.34	0.07	1.04	0.11	0.84	0.12
PC(17:0/20:4)	PC	37:4	+H	795.5778	C45 H82 O8 N1 P1	8.713	3.14	0.23	2.21	0.37	1.92	0.25
PC(15:0/22:6)	PC	37:6	+H	791.5465	C45 H78 O8 N1 P1	7.664	0.63	0.06	0.48	0.05	0.56	0.07
PC(18:0/20:3)	PC	38:3	+H	811.6091	C46 H86 O8 N1 P1	9.657	40.43	2.01	42.65	3.79	40.52	5.69
PC(18:0/20:4)	PC	38:4	+H	809.5935	C46 H84 O8 N1 P1	9.303	69.99	2.91	102.59	4.86	80.53	6.11
PC(18:1/20:4)	PC	38:5	+H	807.5778	C46 H82 O8 N1 P1	8.468	16.00	0.59	1.82	0.46	3.93	0.59
PC(16:0/22:6)	PC	38:6	+H	805.5622	C46 H80 O8 N1 P1	8.157	65.24	3.68	68.85	4.80	70.29	5.24
PC(18:2/20:4)	PC	38:6	+H	805.5622	C46 H80 O8 N1 P1	8.768	70.27	3.78	8.51	0.73	8.62	1.20
PC(20:5/18:2)	PC	38:7	+H	803.5465	C46 H78 O8 N1 P1	8.368	7.65	0.20	0.77	0.11	0.92	0.21
PC(18:3/20:5)	PC	38:8	+H	801.5309	C46 H76 O8 N1 P1	7.527	1.15	0.09	0.75	0.14	0.78	0.25
PC(17:0/22:6)	PC	39:6	+H	819.5778	C47 H82 O8 N1 P1	8.476	0.69	0.06	1.11	0.15	1.27	0.14
PC(18:0/22:4)	PC	40:4	+H	837.6248	C48 H88 O8 N1 P1	9.879	3.98	0.18	3.11	0.45	3.23	0.49
PC(18:0/22:5)	PC	40:5	+H	835.6091	C48 H86 O8 N1 P1	9.084	2.65	0.10	4.15	0.36	4.56	0.35
PC(18:0/22:6)	PC	40:6	+H	833.5935	C48 H84 O8 N1 P1	9.035	21.47	1.54	27.80	2.55	28.44	3.10
PC(18:1/22:6)	PC	40:7	+H	831.5778	C48 H82 O8 N1 P1	8.212	4.50	0.30	0.57	0.08	0.73	0.52
PC(20:1/22:6)	PC	42:7	+H	859.6091	C50 H86 O8 N1 P1	9.06	0.22	0.02	0.42	0.04	0.25	0.03
PC(20:4/22:6)	PC	42:10	+H	853.5622	C50 H80 O8 N1 P1	7.24	0.23	0.02	0.26	0.02	0.24	0.02
PC(16:0e/16:0)	PC	32:0e	+H	719.5829	C40 H82 O7 N1 P1	9.776	2.17	0.10	2.38	0.24	1.89	0.21
PC(16:0e/16:1)	PC	32:1e	+H	717.5672	C40 H80 O7 N1 P1	9.051	1.43	0.08	0.40	0.04	0.45	0.06
PC(18:0e/16:0)	PC	34:0e	+H	747.6142	C42 H86 O7 N1 P1	10.591	0.33	0.02	0.37	0.03	0.29	0.04
PC(16:0e/18:1)	PC	34:1e	+H	743.5829	C42 H82 O7 N1 P1	8.855	5.14	0.31	5.76	0.47	5.27	0.45
PC(16:0e/18:2)	PC	34:2e	+H	743.5829	C42 H82 O7 N1 P1	9.148	4.74	0.31	4.54	0.46	4.20	0.57
PC(16:0p/18:2)	PC	34:2p	+H	741.5672	C42 H80 O7 N1 P1	9.012	6.21	0.34	6.62	0.57	5.77	0.62
PC(18:0p/18:2)	PC	36:2p	+H	769.5985	C44 H84 O7 N1 P1	9.87	5.89	0.27	2.16	0.15	1.65	0.18
PC(16:0e/20:4)	PC	36:4e	+H	767.5829	C44 H82 O7 N1 P1	8.973	14.27	0.52	17.71	1.36	15.38	1.00

PC(16:0p/20:4)	PC	36:4p	+H	765.5672	C44 H80 O7 N1 P1	8.822	7.82	0.29	11.12	0.88	9.52	0.60
PC(16:0p/20:5)	PC	36:5p	+H	763.5516	C44 H78 O7 N1 P1	8.199	0.31	0.04	0.46	0.08	0.53	0.14
PC(18:0e/20:3)	PC	38:3e	+H	797.6298	C46 H88 O7 N1 P1	9.876	1.57	0.07	0.65	0.05	0.54	0.10
PC(18:0e/20:4)	PC	38:4e	+H	795.6142	C46 H86 O7 N1 P1	9.835	9.11	0.37	7.79	0.64	5.27	0.52
PC(18:0p/20:4)	PC	38:4p	+H	793.5985	C46 H84 O7 N1 P1	8.989	12.61	0.44	13.58	1.00	12.14	0.80
PC(18:0e/22:6)	PC	40:6e	+H	819.6142	C48 H86 O7 N1 P1	9.572	1.18	0.07	1.39	0.08	1.19	0.10
PC(18:0p/22:6)	PC	40:6p	+H	817.5985	C48 H84 O7 N1 P1	8.718	1.63	0.11	1.72	0.11	1.70	0.18
PC(18:1p/22:6)	PC	40:7p	+H	815.5829	C48 H82 O7 N1 P1	8.327	1.21	0.04	0.33	0.03	0.30	0.03
PC(32:1)	PC	32:1	+H	803.5465	C46 H78 O8 N1 P1	8.148	4.71	0.27	7.42	0.86	8.95	1.62
PC(36:0)	PC	36:0	+H	789.6248	C44 H88 O8 N1 P1	10.843	0.23	0.01	0.38	0.03	0.33	0.02
PC(36:5)	PC	36:5	+H	779.5465	C44 H78 O8 N1 P1	7.505	10.92	0.18	6.01	0.26	6.33	0.25
PC(40:8)	PC	40:8	+H	829.5622	C48 H80 O8 N1 P1	7.255	1.15	0.06	1.34	0.09	1.23	0.07
PC(42:2p)	PC	42:2p	+H	853.6924	C50 H96 O7 N1 P1	11.088	0.46	0.03	0.31	0.03	0.30	0.04
PE(16:0p/20:4)	PE	36:4p	+H	723.5203	C41 H74 O7 N1 P1	9.094	5.41	0.69	1.29	0.51	2.49	0.44
PE(18:0/20:4)	PE	38:4	+H	767.5465	C43 H78 O8 N1 P1	9.547	6.93	0.57	2.88	0.75	5.00	0.75
PE(18:0p/18:2)	PE	36:2p	+H	727.5516	C41 H78 O7 N1 P1	10.072	2.57	0.29	0.38	0.13	1.17	0.20
PE(18:0p/20:4)	PE	38:4p	+H	751.5516	C43 H78 O7 N1 P1	9.973	12.55	1.21	2.59	0.99	4.59	0.72
PI(16:0/20:4)	PI	36:4	-H	858.5258	C45 H79 O13 N0 P1	7.44	0.80	0.04	0.74	0.47	0.89	0.14
PI(18:0/20:4)	PI	38:4	-H	886.5571	C47 H83 O13 N0 P1	8.316	7.58	0.33	5.39	4.19	5.83	0.69
PI(18:0/22:6)	PI	40:6	-H	910.5571	C49 H83 O13 N0 P1	8.058	0.29	0.03	0.18	0.15	0.26	0.06
PI(18:1/20:4)	PI	38:5	-H	884.5415	C47 H81 O13 N0 P1	7.498	0.52	0.03	0.39	0.23	0.45	0.04
PS(40:4p)	PS	40:4p	-H	823.5727	C46 H82 O9 N1 P1	8.823	2.77	0.22	3.64	2.93	2.24	0.47
SM(d17:1/16:0)	SM	d33:1	+H	688.5519	C38 H77 O6 N2 P1	7.887	6.92	0.44	8.33	0.61	7.42	0.66
SM(d16:0/18:1)	SM	d34:1	+H	702.5676	C39 H79 O6 N2 P1	8.37	87.66	2.07	119.45	8.38	103.19	6.53
SM(d18:0/18:1)	SM	d36:1	+H	730.5989	C41 H83 O6 N2 P1	9.343	16.59	0.65	26.04	1.53	20.39	1.47
SM(d14:1/22:3)	SM	d36:4	+H	724.5519	C41 H77 O6 N2 P1	8.364	6.37	0.15	3.20	0.23	3.00	0.21
SM(d18:2/18:3)	SM	d36:5	+H	722.5363	C41 H75 O6 N2 P1	7.504	2.35	0.11	1.95	0.29	1.90	0.18
SM(d22:0/16:0)	SM	d38:0	+H	760.6458	C43 H89 O6 N2 P1	10.491	2.22	0.11	1.04	0.11	0.58	0.10
SM(d20:1/18:1)	SM	d38:2	+H	756.6145	C43 H85 O6 N2 P1	9.418	5.88	0.31	9.15	0.63	7.65	0.51
SM(d18:1/20:3)	SM	d38:4	+H	752.5832	C43 H81 O6 N2 P1	9.354	1.23	0.05	0.76	0.04	0.47	0.04
SM(d20:0/20:3)	SM	d40:3	+H	782.6302	C45 H87 O6 N2 P1	9.387	1.69	0.11	2.25	0.26	1.96	0.16
SM(d20:1/20:3)	SM	d40:4	+H	780.6145	C45 H85 O6 N2 P1	10.202	2.75	0.15	2.83	0.22	2.41	0.14

SM(d15:0/26:1)	SM	d41:1	+H	800.6771	C46 H93 O6 N2 P1	11.312	18.35	1.03	21.88	1.43	18.26	1.69
SM(d22:1/19:1)	SM	d41:2	+H	798.6615	C46 H91 O6 N2 P1	10.523	16.57	1.02	19.74	1.41	13.45	1.50
SM(d17:1/24:3)	SM	d41:4	+H	794.6302	C46 H87 O6 N2 P1	10.597	0.97	0.08	1.07	0.12	0.79	0.11
SM(d18:1/24:3)	SM	d42:4	+H	808.6458	C47 H89 O6 N2 P1	10.97	6.33	0.23	6.66	0.30	5.40	0.49
SM(d18:1/26:3)	SM	d44:4	+H	836.6771	C49 H93 O6 N2 P1	11.666	4.44	0.21	6.47	0.47	3.98	0.53
SM(d30:1)	SM	d30:1	+H	646.505	C35 H71 O6 N2 P1	6.352	0.38	0.04	0.51	0.04	0.39	0.05
SM(d31:1)	SM	d31:1	+H	660.5206	C36 H73 O6 N2 P1	6.862	0.24	0.03	0.20	0.02	0.20	0.02
SM(d32:0)	SM	d32:0	+H	676.5519	C37 H77 O6 N2 P1	7.759	0.40	0.02	0.50	0.05	0.35	0.04
SM(d32:1)	SM	d32:1	+H	674.5363	C37 H75 O6 N2 P1	7.38	10.54	0.70	12.97	1.07	10.32	0.93
SM(d32:2)	SM	d32:2	+H	672.5206	C37 H73 O6 N2 P1	6.462	1.40	0.09	1.66	0.14	1.42	0.16
SM(d34:0)	SM	d34:0	+H	704.5832	C39 H81 O6 N2 P1	8.749	4.30	0.21	8.48	0.52	5.56	0.53
SM(d34:2)	SM	d34:2	+H	700.5519	C39 H77 O6 N2 P1	7.261	21.23	1.03	22.18	2.52	19.67	1.62
SM(d34:4)	SM	d34:4	+H	696.5206	C39 H73 O6 N2 P1	7.382	1.20	0.09	1.20	0.13	0.85	0.10
SM(d34:5)	SM	d34:5	+H	694.505	C39 H71 O6 N2 P1	6.474	0.20	0.02	0.37	0.03	0.29	0.03
SM(d35:4)	SM	d35:4	+H	710.5363	C40 H75 O6 N2 P1	7.887	0.76	0.05	0.68	0.05	0.63	0.05
SM(d36:2)	SM	d36:2	+H	728.5832	C41 H81 O6 N2 P1	8.221	7.54	0.29	11.71	0.66	10.28	0.64
SM(d36:3)	SM	d36:3	+H	726.5676	C41 H79 O6 N2 P1	8.765	0.97	0.07	1.02	0.09	0.84	0.07
SM(d39:1)	SM	d39:1	+H	772.6458	C44 H89 O6 N2 P1	10.601	7.26	0.61	9.71	0.86	7.11	0.93
SM(d40:0)	SM	d40:0	+H	788.6771	C45 H93 O6 N2 P1	10.935	3.96	0.16	1.91	0.17	0.93	0.17
SM(d40:1)	SM	d38:1	+H	758.6302	C43 H87 O6 N2 P1	9.907	20.05	1.13	68.39	4.73	44.63	5.83
SM(d40:2)	SM	d40:2	+H	784.6458	C45 H89 O6 N2 P1	9.866	32.55	1.63	38.97	3.82	28.78	3.05
SM(d42:1)	SM	d42:1	+H	814.6928	C47 H95 O6 N2 P1	11.662	26.85	1.23	11.15	0.74	8.65	0.71
SM(d42:1+pO)	SM	d42:1+pO	+H	830.6877	C47 H95 O7 N2 P1	10.504	0.57	0.03	0.70	0.06	0.53	0.05
SM(d42:2)	SM	d42:2	+H	812.6771	C47 H93 O6 N2 P1	10.588	85.79	3.95	135.16	8.82	115.52	10.20
SM(d42:3)	SM	d42:3	+H	810.6615	C47 H91 O6 N2 P1	9.923	42.34	2.12	54.98	5.40	52.50	3.77
SM(d42:5)	SM	d42:5	+H	806.6302	C47 H87 O6 N2 P1	9.852	4.83	0.23	4.35	0.35	3.74	0.33
SM(d43:2)	SM	d43:2	+H	826.6928	C48 H95 O6 N2 P1	11.105	4.06	0.23	5.17	0.49	3.15	0.38
SM(d43:4)	SM	d43:4	+H	822.6615	C48 H91 O6 N2 P1	11.321	2.96	0.16	3.14	0.22	2.37	0.27
SM(d44:5)	SM	d44:5	+H	834.6615	C49 H91 O6 N2 P1	10.891	13.38	0.55	12.90	0.78	12.19	0.78
SM(d44:6)	SM	d44:6	+H	832.6458	C49 H89 O6 N2 P1	10.213	6.14	0.27	5.60	0.39	5.36	0.31
TG(44:1)	TG	44:1	+NH4	748.6581	C47 H88 O6	12.444	1.05	0.42	2.32	1.86	1.35	2.41
TG(44:2)	TG	44:2	+NH4	746.6424	C47 H86 O6	12.331	0.49	0.22	1.26	1.14	0.79	1.52

TG(45:1)	TG	45:1	+NH4	762.6737	C48 H90 O6	12.433	0.35	0.05	0.34	0.14	0.43	0.23
TG(45:2)	TG	45:2	+NH4	760.6581	C48 H88 O6	12.333	0.26	0.03	0.33	0.14	0.33	0.30
TG(46:0)	TG	46:0	+NH4	778.705	C49 H94 O6	12.657	0.80	0.25	1.95	0.83	1.38	1.88
TG(46:1)	TG	46:1	+NH4	776.6894	C49 H92 O6	12.547	2.42	0.50	4.60	2.46	3.77	4.26
TG(46:2)	TG	46:2	+NH4	774.6737	C49 H90 O6	12.387	2.34	0.50	4.81	2.59	2.85	3.89
TG(46:3)	TG	46:3	+NH4	772.6581	C49 H88 O6	12.345	0.65	0.18	1.56	0.96	1.08	1.82
TG(46:4)	TG	46:4	+NH4	770.6424	C49 H86 O6	12.163	0.12	0.03	0.35	0.22	0.25	0.29
TG(47:1)	TG	47:1	+NH4	790.705	C50 H94 O6	12.529	0.72	0.07	0.66	0.21	0.67	0.38
TG(47:2)	TG	47:2	+NH4	788.6894	C50 H92 O6	12.438	0.63	0.07	0.73	0.23	0.66	0.34
TG(48:0)	TG	48:0	+NH4	806.7363	C51 H98 O6	12.757	2.57	0.53	4.41	2.44	3.48	4.03
TG(48:1)	TG	48:1	+NH4	804.7207	C51 H96 O6	12.647	8.17	0.97	13.50	5.14	11.18	10.34
TG(48:2)	TG	48:2	+NH4	802.705	C51 H94 O6	12.487	9.23	1.21	16.60	5.86	13.88	9.35
TG(48:3)	TG	48:3	+NH4	800.6894	C51 H92 O6	12.398	4.36	0.74	8.47	3.79	6.73	5.05
TG(48:4)	TG	48:4	+NH4	798.6737	C51 H90 O6	12.363	1.19	0.20	2.52	1.51	1.99	1.91
TG(48:5)	TG	48:5	+NH4	796.6581	C51 H88 O6	12.26	0.18	0.03	0.54	0.29	0.58	0.41
TG(49:1)	TG	49:1	+NH4	818.7363	C52 H98 O6	12.685	2.05	0.22	1.98	0.64	2.24	0.91
TG(49:2)	TG	49:2	+NH4	816.7207	C52 H96 O6	12.534	1.70	0.20	2.12	0.47	2.10	0.80
TG(49:3)	TG	49:3	+NH4	814.705	C52 H94 O6	12.505	0.83	0.10	1.26	0.29	1.19	0.40
TG(50:1)	TG	50:1	+NH4	832.752	C53 H100 O6	12.746	30.00	4.62	36.55	15.16	46.61	38.83
TG(50:2)	TG	50:2	+NH4	830.7363	C53 H98 O6	12.646	35.82	3.95	61.08	15.59	53.66	26.05
TG(50:3)	TG	50:3	+NH4	828.7207	C53 H96 O6	12.498	23.39	3.04	37.98	8.82	43.20	14.85
TG(50:4)	TG	50:4	+NH4	826.705	C53 H94 O6	12.417	9.34	1.27	14.47	5.20	14.50	6.52
TG(50:5)	TG	50:5	+NH4	824.6894	C53 H92 O6	12.391	1.93	0.23	3.45	1.78	4.36	2.68
TG(50:6)	TG	50:6	+NH4	822.6737	C53 H90 O6	12.261	0.27	0.04	0.92	0.45	1.25	0.74
TG(51:1)	TG	51:1	+NH4	846.7676	C54 H102 O6	12.777	2.19	0.34	2.37	0.88	2.84	1.12
TG(51:2)	TG	51:2	+NH4	844.752	C54 H100 O6	12.63	4.45	0.57	4.77	1.81	7.07	1.41
TG(51:3)	TG	51:3	+NH4	842.7363	C54 H98 O6	12.596	3.56	0.52	4.31	0.84	5.89	0.94
TG(51:4)	TG	51:4	+NH4	840.7207	C54 H96 O6	12.51	1.94	0.27	2.18	0.47	2.92	0.45
TG(52:1)	TG	52:1	+NH4	860.7833	C55 H104 O6	12.761	27.37	4.17	30.57	11.51	39.55	20.12
TG(52:2)	TG	52:2	+NH4	858.7676	C55 H102 O6	12.737	149.63	20.55	151.46	43.25	231.98	49.24
TG(52:2)	TG	52:2	+NH4	852.7207	C55 H96 O6	12.433	16.47	2.28	26.14	7.64	33.02	8.78
TG(52:3)	TG	52:3	+NH4	856.752	C55 H100 O6	12.648	140.93	15.57	154.40	28.90	223.83	37.68

TG(52:4)	TG	52:4	+NH4	854.7363	C55 H98 O6	12.559	64.98	10.29	77.64	14.86	103.50	21.24
TG(52:6)	TG	52:6	+NH4	850.705	C55 H94 O6	12.409	3.36	0.42	7.16	2.76	7.35	3.88
TG(52:7)	TG	52:7	+NH4	848.6894	C55 H92 O6	12.276	0.49	0.08	1.47	0.68	1.76	1.16
TG(53:2)	TG	53:2	+NH4	872.7833	C56 H104 O6	12.772	4.13	0.60	4.10	1.17	5.82	0.86
TG(53:3)	TG	53:3	+NH4	870.7676	C56 H102 O6	12.684	4.09	0.44	4.39	0.96	5.69	0.66
TG(53:4)	TG	53:4	+NH4	868.752	C56 H100 O6	12.599	2.31	0.28	2.82	0.44	3.24	0.47
TG(53:5)	TG	53:5	+NH4	866.7363	C56 H98 O6	12.523	1.08	0.13	1.75	0.32	2.26	0.35
TG(54:1)	TG	54:1	+NH4	888.8146	C57 H108 O6	12.917	5.03	0.85	4.48	1.73	5.56	3.25
TG(54:2)	TG	54:2	+NH4	886.7989	C57 H106 O6	12.831	22.77	4.04	24.26	6.80	32.37	7.18
TG(54:3)	TG	54:3	+NH4	884.7833	C57 H104 O6	13.036	62.51	8.87	60.54	12.79	70.84	9.14
TG(54:4)	TG	54:4	+NH4	882.7676	C57 H102 O6	12.648	55.36	6.20	66.38	8.89	76.41	11.15
TG(54:5)	TG	54:5	+NH4	880.752	C57 H100 O6	12.568	36.36	5.51	57.03	10.10	60.18	11.23
TG(54:6)	TG	54:6	+NH4	878.7363	C57 H98 O6	12.455	19.58	2.83	33.33	8.99	41.87	9.42
TG(54:7)	TG	54:7	+NH4	876.7207	C57 H96 O6	12.438	5.63	0.92	13.08	4.41	13.75	6.04
TG(54:8)	TG	54:8	+NH4	874.705	C57 H94 O6	12.354	0.91	0.15	2.93	1.05	2.38	1.70
TG(56:2)	TG	56:2	+NH4	914.8302	C59 H110 O6	12.907	1.68	0.24	1.29	0.35	1.53	0.58
TG(56:3)	TG	56:3	+NH4	912.8146	C59 H108 O6	12.827	2.31	0.31	2.89	0.55	2.79	0.50
TG(56:4)	TG	56:4	+NH4	910.7989	C59 H106 O6	12.734	3.96	0.39	5.50	0.79	5.16	0.97
TG(56:5)	TG	56:5	+NH4	908.7833	C59 H104 O6	12.685	10.09	1.03	17.28	1.89	14.36	2.12
TG(56:6)	TG	56:6	+NH4	906.7676	C59 H102 O6	12.599	15.74	1.71	29.39	3.02	22.68	4.25
TG(56:7)	TG	56:7	+NH4	904.752	C59 H100 O6	12.54	16.68	2.34	31.04	6.65	27.02	8.16
TG(56:8)	TG	56:8	+NH4	902.7363	C59 H98 O6	12.464	8.73	1.64	17.99	5.84	15.98	6.37
TG(56:9)	TG	56:9	+NH4	900.7207	C59 H96 O6	12.319	1.38	0.29	4.71	1.69	3.82	2.16
TG(56:10)	TG	56:10	+NH4	898.705	C59 H94 O6	12.23	0.18	0.04	0.81	0.45	0.50	0.50
TG(58:1)	TG	58:1	+NH4	944.8772	C61 H116 O6	13.11	0.55	0.18	0.24	0.19	0.45	0.25
TG(58:6)	TG	58:6	+NH4	934.7989	C61 H106 O6	12.673	1.49	0.13	2.70	0.30	2.08	0.44
TG(58:7)	TG	58:7	+NH4	932.7833	C61 H104 O6	12.566	2.64	0.29	5.09	0.69	4.80	1.01
TG(58:8)	TG	58:8	+NH4	930.7676	C61 H102 O6	12.556	4.32	0.56	8.89	1.46	6.37	2.11
TG(58:9)	TG	58:9	+NH4	928.752	C61 H100 O6	12.475	3.66	0.55	10.07	2.42	6.97	2.33
TG(58:10)	TG	58:10	+NH4	926.7363	C61 H98 O6	12.401	1.55	0.30	7.19	1.83	3.95	1.75
TG(58:11)	TG	58:11	+NH4	924.7207	C61 H96 O6	12.248	0.29	0.09	1.54	1.10	1.24	1.23
TG(60:10)	TG	60:10	+NH4	954.7676	C63 H102 O6	12.445	0.68	0.09	2.02	0.42	1.44	0.48

TG(60:12)	TG	60:12	+NH4	950.7363	C63 H98 O6	12.374	0.35	0.12	1.52	1.11	1.13	0.87
TG(62:13)	TG	62:13	+NH4	976.752	C65 H100 O6	12.345	0.11	0.05	0.66	0.42	0.27	0.22

Table S2: Lipid classes are reported as Median [Interquartile Range (Q1-Q3)] in the three groups examined. Fold change (FC) values are reported for the comparisons between patients with Heterozygous Familial Hypercholesterolemia (HeFH) versus healthy controls (CTR), patients without Pathogenic/ Uncertain Significance variants (FH/V−/USV−) versus CTR, and HeFH versus FH/V−/USV−. (***Bolded FC values*** indicate classes with statistically significant differences ($p < 0.05$) as determined by the Kruskal-Wallis test with FDR correction)

	CTR		HeFH		FH/V−/USV−					
**Class**	**Median (µg/mL)**	**IQR**	**Median (µg/mL)**	**IQR**	**Median (µg/mL)**	**IQR**	**adjusted p**	**FC (HeFH vs CTR)**	**FC (FH/V−/USV− vs CTR)**	**FC (HeFH vs FH/V−/USV−)**
Cer	6.19	5.33–7.49	8.89	7.55–11.45	10.61	7.12–17.69	0.0001	**1.44**	**1.71**	-1.19
ChE	5678.69	4983.82–6071.68	8701.79	6400.97–10138.67	8083.91	5843.25–11819.30	0.0001	**1.53**	**1.42**	1.08
DG	23.11	17.36–30.09	20.48	13.10–62.17	29.94	19.09–38.98	0.2847	-1.13	1.30	-1.46
LPC	71.98	63.99–84.14	93.33	74.99–133.48	71.34	67.15–97.24	0.0191	**1.30**	-1.01	1.31
PC	826.07	738.75–930.81	867.29	833.51–1016.20	912.78	783.68–1009.26	0.1526	1.05	1.10	-1.05
PI	9.17	8.22–10.43	6.92	4.70–9.30	6.94	4.49–8.84	0.0185	**-1.32**	**-1.32**	-1.00
PE	27.69	22.31–37.54	7.15	0.61–22.46	16.34	11.22–20.68	0.0001	**-3.87**	**-1.69**	-2.29
SM	480.83	441.37–705.34	648.50	520.51–825.43	525.36	395.36–695.30	0.0036	**1.35**	1.09	**1.23**
TG	903.14	616.76–1083.63	1130.09	832.52–1753.38	1358.15	877.22–2161.79	0.0170	**1.25**	**1.50**	-1.20

Table S3: Correlation results (Spearman R and p-value) between lipid classes and clinical data in the overall cohorts, control (CTR), HeFH, and FH/V−/USV−.

Overall	Genotype		Sex		Age		LDL-c mg/dL		Tot Chol mg/dL		HDL-c mg/dL		NonHDL Chol mg/dL		TG mg/dL	
	Spearman R	p-value	Spearman R	p-value	Spearman R	p-value	Spearman R	p-value	Spearman R	p-value	Spearman R	p-value	Spearman R	p-value	Spearman R	p-value
**Cer**	0.48	0.0001	-0.05	0.6819	0.17	0.2030	0.53	< 0.0001	0.58	< 0.0001	-0.06	0.6682	0.63	< 0.0001	0.36	0.0050
**ChE**	0.51	< 0.0001	0.09	0.4940	0.17	0.2018	0.55	< 0.0001	0.57	< 0.0001	0.18	0.1731	0.51	< 0.0001	-0.09	0.5018
**DG**	-0.11	0.3799	-0.29	0.0246	0.23	0.0812	-0.02	0.8652	0.00	0.9931	-0.48	0.0001	0.14	0.2903	0.82	< 0.0001
**LPC**	0.36	0.0044	-0.07	0.5845	-0.10	0.4308	0.27	0.0332	0.31	0.0153	-0.06	0.6561	0.32	0.0131	0.15	0.2464
**PC**	0.24	0.0587	0.15	0.2339	-0.07	0.6184	0.56	< 0.0001	0.75	< 0.0001	0.28	0.0312	0.64	< 0.0001	0.24	0.0627
**PE**	-0.59	< 0.0001	0.29	0.0246	0.02	0.9050	-0.27	0.0362	-0.24	0.0579	0.38	0.0026	-0.35	0.0064	-0.25	0.0560
**PI**	-0.32	0.0125	-0.04	0.7384	0.02	0.8986	0.03	0.8471	0.05	0.6977	0.05	0.7235	0.00	0.9990	0.25	0.0485
**SM**	0.42	0.0007	0.15	0.2462	-0.09	0.5075	0.65	< 0.0001	0.80	< 0.0001	0.26	0.0420	0.72	< 0.0001	0.09	0.5137
**TG**	0.23	0.0710	-0.34	0.0079	0.25	0.0498	0.12	0.3474	0.17	0.1972	-0.56	< 0.0001	0.35	0.0061	0.97	< 0.0001

CTR	Sex		Age		LDL-c mg/dL		Tot Chol mg/dL		HDL-c mg/dL		NonHDL Chol mg/dL		TG mg/dL	
	Spearman R	p-value	Spearman R	p-value	Spearman R	p-value	Spearman R	p-value	Spearman R	p-value	Spearman R	p-value	Spearman R	p-value
**Cer**	-0.29	0.1912	0.0209	0.9264	0.55	0.0084	0.27	0.2207	-0.28	0.2086	0.53	0.0111	0.30	0.1692
**ChE**	0.31	0.1666	0.0124	0.9562	0.33	0.1369	0.69	0.0003	0.63	0.0017	0.39	0.0733	-0.12	0.6026
**DG**	-0.03	0.8870	0.3613	0.0985	0.32	0.1503	0.04	0.8550	-0.52	0.0139	0.41	0.0556	0.88	< 0.0001
**LPC**	-0.48	0.0229	-0.0164	0.9423	0.12	0.5795	-0.02	0.9262	0.14	0.5250	0.04	0.8612	-0.04	0.8515
**PC**	0.58	0.0047	0.1278	0.5709	0.03	0.9006	0.53	0.0108	0.70	0.0003	0.15	0.4978	0.01	0.9682
**PE**	0.11	0.6178	0.1233	0.5847	-0.38	0.0831	-0.17	0.4602	0.47	0.0274	-0.32	0.1415	-0.32	0.1531
**PI**	0.48	0.0229	0.3110	0.1589	-0.16	0.4690	0.06	0.7983	0.52	0.0127	-0.07	0.7567	-0.03	0.9027
**SM**	0.39	0.0759	0.0119	0.9582	0.23	0.3053	0.65	0.0010	0.62	0.0021	0.29	0.1897	-0.08	0.7171
**TG**	-0.18	0.4308	0.3834	0.0782	0.38	0.0846	0.13	0.5700	-0.49	0.0216	0.48	0.0254	0.95	< 0.0001

FH/V-/USV-	Sex		Age		LDL-c mg/dL		Tot Chol mg/dL		HDL-c mg/dL		NonHDL Chol mg/dL		TG mg/dL	
	Spearman R	p-value	Spearman R	p-value	Spearman R	p-value	Spearman R	p-value	Spearman R	p-value	Spearman R	p-value	Spearman R	p-value
**Cer**	0.26	0.2844	-0.3812	0.1073	0.75	0.0002	0.80	< 0.0001	0.17	0.4972	0.82	< 0.0001	0.50	0.0305
**ChE**	0.26	0.2844	-0.1853	0.4475	0.55	0.0157	0.50	0.0299	0.34	0.1535	0.42	0.0725	-0.42	0.0712
**DG**	-0.40	0.0911	0.2942	0.2214	-0.13	0.5963	-0.05	0.8417	-0.51	0.0242	0.09	0.7049	0.83	< 0.0001
**LPC**	0.16	0.5146	-0.0307	0.9006	0.36	0.1265	0.48	0.0380	0.10	0.6888	0.41	0.0814	0.16	0.5090
**PC**	0.24	0.3243	-0.3680	0.1211	0.76	0.0002	0.91	< 0.0001	0.38	0.1102	0.81	< 0.0001	0.28	0.2457
**PE**	0.52	0.0231	0.1089	0.6572	0.15	0.5280	0.30	0.2065	0.55	0.0154	0.15	0.5494	-0.16	0.5160
**PI**	-0.32	0.1835	0.2047	0.4007	0.01	0.9545	0.04	0.8810	-0.30	0.2187	0.10	0.6757	0.66	0.0020
**SM**	0.26	0.2844	-0.1924	0.4301	0.71	0.0007	0.84	< 0.0001	0.42	0.0717	0.75	0.0002	0.21	0.3868
**TG**	-0.26	0.2844	0.1177	0.6313	0.11	0.6680	0.16	0.5233	-0.44	0.0612	0.33	0.1641	0.98	< 0.0001

HeFH	Sex		Age		LDL-c mg/dL		Tot Chol mg/dL		HDL-c mg/dL		NonHDL Chol mg/dL		TG mg/dL	
	Spearman R	p-value	Spearman R	p-value	Spearman R	p-value	Spearman R	p-value	Spearman R	p-value	Spearman R	p-value	Spearman R	p-value
**Cer**	-0.06	0.7899	0.2040	0.3883	0.68	0.0009	0.65	0.0018	0.29	0.2071	0.63	0.0030	-0.11	0.6313
**ChE**	0.03	0.9091	0.3116	0.1811	0.61	0.0045	0.57	0.0081	0.38	0.1029	0.46	0.0401	-0.65	0.0018
**DG**	-0.35	0.1251	-0.1596	0.5016	0.21	0.3830	0.25	0.2898	-0.47	0.0377	0.39	0.0851	0.92	< 0.0001
**LPC**	-0.03	0.9091	-0.1069	0.6538	0.09	0.7072	0.13	0.5955	-0.09	0.6998	0.17	0.4692	0.07	0.7671
**PC**	-0.30	0.1988	-0.1355	0.5690	0.69	0.0007	0.73	0.0003	-0.01	0.9774	0.75	0.0001	0.35	0.1263
**PE**	0.28	0.2287	0.0128	0.9573	-0.54	0.0136	-0.53	0.0156	-0.11	0.6441	-0.53	0.0158	0.20	0.4050
**PI**	-0.25	0.2970	-0.1355	0.5690	0.33	0.1577	0.45	0.0474	-0.08	0.7521	0.47	0.0380	0.61	0.0042
**SM**	0.01	0.9697	-0.0625	0.7936	0.78	0.0001	0.79	0.0000	0.28	0.2311	0.71	0.0004	-0.20	0.4031
**TG**	-0.43	0.0603	-0.2296	0.3302	-0.05	0.8451	-0.02	0.9198	-0.60	0.0050	0.16	0.4892	0.95	< 0.0001

Table S4: Area Under the Curve (AUC) and 95% Confidence interval of the significant Sphingomyelin (SM) species

Lipid	AUC	95% Confidence interval
SM(d32:0)	0.7184	0.5582 - 0.8787
SM(d38:4)	0.8079	0.6634 - 0.9524
SM(d43:2)	0.7737	0.6281 - 0.9192
SM(d44:4)	0.7315	0.5695 - 0.8937
SM (total)	0.6921	0.5260 - 0.8582
